# Molecular Recognition in Complexes of TRF Proteins with Telomeric DNA

**DOI:** 10.1371/journal.pone.0089460

**Published:** 2014-02-26

**Authors:** Miłosz Wieczór, Adrian Tobiszewski, Paweł Wityk, Bartłomiej Tomiczek, Jacek Czub

**Affiliations:** 1 Department of Physical Chemistry, Faculty of Chemistry, Gdansk University of Technology, Gdansk, Poland; 2 Department of Genetics, Evolution and Environment, University College London, London, United Kingdom; Weizmann Institute of Science, Israel

## Abstract

Telomeres are specialized nucleoprotein assemblies that protect the ends of linear chromosomes. In humans and many other species, telomeres consist of tandem TTAGGG repeats bound by a protein complex known as shelterin that remodels telomeric DNA into a protective loop structure and regulates telomere homeostasis. Shelterin recognizes telomeric repeats through its two major components known as Telomere Repeat-Binding Factors, TRF1 and TRF2. These two homologous proteins are therefore essential for the formation and normal function of telomeres. Indeed, TRF1 and TRF2 are implicated in a plethora of different cellular functions and their depletion leads to telomere dysfunction with chromosomal fusions, followed by apoptotic cell death. More specifically, it was found that TRF1 acts as a negative regulator of telomere length, and TRF2 is involved in stabilizing the loop structure. Consequently, these proteins are of great interest, not only because of their key role in telomere maintenance and stability, but also as potential drug targets. In the current study, we investigated the molecular basis of telomeric sequence recognition by TRF1 and TRF2 and their DNA binding mechanism. We used molecular dynamics (MD) to calculate the free energy profiles for binding of TRFs to telomeric DNA. We found that the predicted binding free energies were in good agreement with experimental data. Further, different molecular determinants of binding, such as binding enthalpies and entropies, the hydrogen bonding pattern and changes in surface area, were analyzed to decompose and examine the overall binding free energies at the structural level. With this approach, we were able to draw conclusions regarding the consecutive stages of sequence-specific association, and propose a novel aspartate-dependent mechanism of sequence recognition. Finally, our work demonstrates the applicability of computational MD-based methods to studying protein-DNA interactions.

## Introduction

Telomeres are complex nucleoprotein structures that serve as protecting and stabilizing agents at the ends of linear chromosomes. Telomeres have evolved in eukaryotes (and some Prokaryota species) to serve as a buffer protecting against the end-replication problem, which stems from the inability of DNA polymerase complex to synthesize the 5′-end of the lagging strand after removal of the most distal RNA primer. In human and other vertebrates, as well as in many other species, telomeric DNA is composed of double-stranded (ds) tandem repeats of the sequence 5′-TTAGGG-3′ ending in a 50–500 nucleotides long single-stranded (ss) 3′ overhang [1]. Telomeric DNA is capable of forming lasso-like structures called telomere loops (T-loops) [2], [3], resulting from the invasion of the double stranded repeat tracts by the 3′ overhang and the consequent formation of a displacement loop (D-loop). Such a structure, in which the natural end of the chromosome is protected by looping back into the duplex telomeric region, is promoted and stabilized by a multiprotein complex, the shelterin [4].

Telomere repeat sequences undergo progressive shortening with each round of replication, thereby protecting the coding regions in genomic DNA from erosion when cells divide. Highly proliferative cells (including stem cells, germ line cells and numerous tumor cell types) express telomerase, a ribonucleoprotein that uses its own internal RNA template to catalyze the addition of telomere repeats onto the 3′ ends of chromosomes, thus maintaining the telomere length [5]. A main purpose of the shelterin capping complex is to protect genome integrity through preventing activation of DNA damage response by the ends of linear chromosomes which would naturally appear as double-strand breaks [6]. Loss of shelterin components has been shown to induce chromosomal aberrations [7], either by DNA ligase IV-mediated NHEJ (non-homologous end joining) repair pathways [8] or by direct activation of the ATM-dependent DNA damage signaling pathway [9]. Consequently, telomere uncapping would eventually lead to cellular senescence or apoptosis. For the above reasons, telomeres and telomere maintenance mechanism still attract a lot of attention as both potential drug targets and aging-related markers [10], [11].

The shelterin itself consists of several different proteins which interact with telomeric DNA in a complex way to shape the structure of the telomere and regulate its maintenance [4]. Two Telomere Repeat-binding Factors, TRF1 and TRF2, which bind specifically to double-stranded telomeric DNA and the POT1 protein, which recognizes the 3′ overhang, have been found [12] to recruit other proteins, namely Rap1, TPP1 and TIN2 to form a telomere-localized shelterin complex [4]. The TRF proteins are therefore largely responsible for the DNA-binding affinity and sequence specificity of the shelterin. Both TRF proteins contain an N-terminal domain responsible for homodimerization (TRF1 and TRF2 do not heterodimerize) and for binding other shelterin proteins, and a C-terminal DNA-binding domain (DBD) similar to the Myb DNA-binding motif [13], [14], both connected through a long, presumably unstructured linker region. Interestingly, despite the high sequence and structural similarity, the TRF proteins have been shown to mediate distinct biological functions. Indeed, their overexpression or deficiency trigger different cellular responses, and their dimerization domains recognize different shelterin-associated factors [2], [14]–[16].

**Figure 1:**
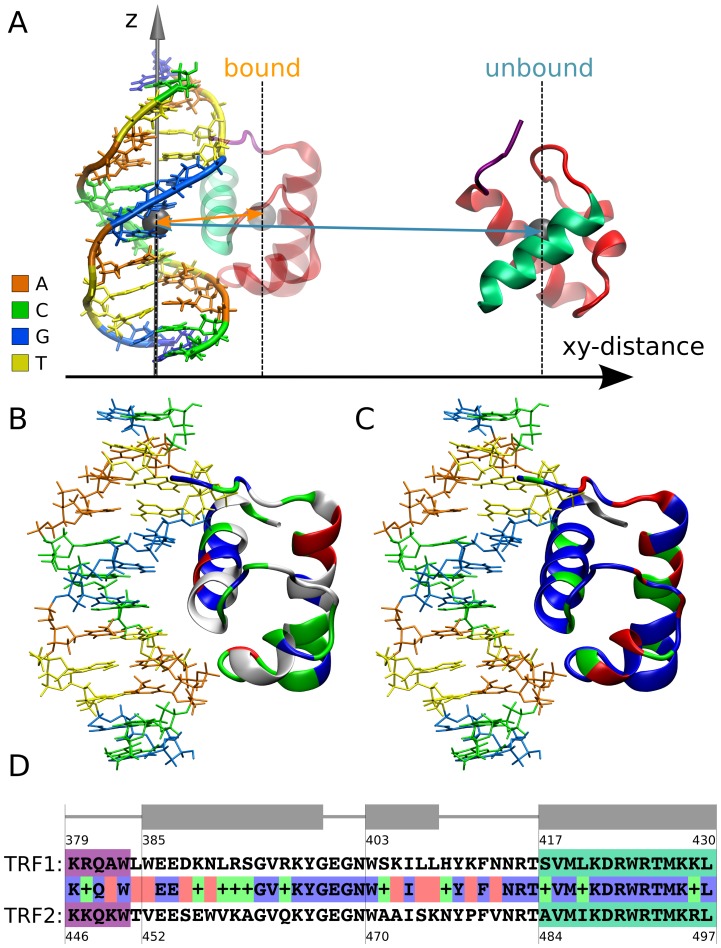
TRF-DNA complex structure and comparison of the TRF1/TRF2 DBD domains. (A) A schematic representation of the system used for the calculation of the binding free energy profiles. The DNA dodecamer (5′-GGTTAGGGTTAG-3′) is aligned with the z axis, and the xy-distance between the centers of mass of TRF and DNA serves as a convenient reaction coordinate describing the binding process (for a precise definition of the reaction coordinate, see Methods and Fig. S1). The C-terminal recognition helix interacting with the DNA major groove is shown in green and the N-terminal linker binding within the minor groove is in purple. (B) Distribution of different types of amino acid residues in the TRF2 structure: hydrophobic residues are depicted in white, hydrophilic in green, basic in blue and acidic in red. (C) Differences in amino acid sequence between TRF1 and TRF2 mapped onto the TRF2 structure in a color coded manner: identical amino acids are marked in blue, green denotes conservative substitutions (little change) and red non-conservative substitutions (significant change). (D) Alignment of TRF1 and TRF2 sequences. Color-coding is consistent with panel C. The purple and green highlights denote the N-terminal linker and the C-terminal helix, respectively.

The DNA-binding domain of the TRF proteins is a tri-helical bundle with the C-terminal helix fitting into the DNA major groove and the N-terminal end, in the linker region, binding to the minor groove (Fig. 1). The NMR [17], [18] and X-ray [19] structures of the TRF1 and TRF2 DBDs (which for simplicity will be referred to as TRF1 and TRF2) bound to ds telomeric DNA have been solved, providing insight into the molecular basis of recognition and binding specificity. DNA-binding constants determined for TRF1 and TRF2 and their several mutants by means of surface plasmon resonance allowed to identify a number of residues important for specific interactions with telomeric DNA [18]. However, macromolecular binding is essentially a dynamic process, and its overall thermodynamic and kinetic description (e.g., experimental binding and rate constants) cannot be rigorously explained at the molecular level using only few microscopic snapshots from structural studies. Rather, such an explanation requires adequate sampling of the equilibrium ensemble along the binding pathway and including the effect of thermally-induced structural fluctuations of the individual residues and solvent at the flexible recognition interface. So far, however, no such detailed information on the binding mechanism of the TRF proteins to DNA is available, which necessarily limits our understanding of the origins of telomere structural stability. Moreover, certain G-triplet specific intercalators were found to disrupt the specific interaction of TRFs with telomeric DNA, which in turn was shown to trigger apoptosis [20], [21]. This suggests that a deeper insight into the molecular determinants of specificity in the TRF-DNA complexes could facilitate further development of potential therapeutics.

In the following study, we examine the molecular basis of sequence-specific affinity of the TRF proteins for telomeric DNA and provide a dynamic picture of the binding process. In order to gain quantitative insight into the mechanisms of DNA recognition by TRFs, the atomistic umbrella sampling simulations are used to determine the free energy profiles for the association of TRF1 and TRF2 with telomere 5′-TTAGGG-3′ DNA repeats. With this approach we obtain the absolute and relative binding free energies which are consistent with the reported experimental values. By analyzing different molecular determinants of the association process, including the hydrogen bonding pattern, binding energies, entropies for single amino acids and changes in surface area, we further decompose the overall free energy profiles and interpret the individual contributions at the structural level. This treatment enables us to examine in detail the consecutive stages of sequence-specific association of the TRF proteins with DNA. Based on this description, we propose a novel, to our knowledge, mechanism of DNA sequence recognition, in which specificity is largely determined by a single aspartate residue of the recognition helix with some contribution of the flanking amino acids. Also discussed are the factors underlying the differences in affinity for DNA observed between TRF1 and TRF2. Additionally, our current results demonstrate that free energy computation methods can be a valuable tool for studying protein-DNA interactions.

## Results and Discussion

### Free Energy of Binding of the TRF Proteins to Telomeric DNA

**Figure 2:**
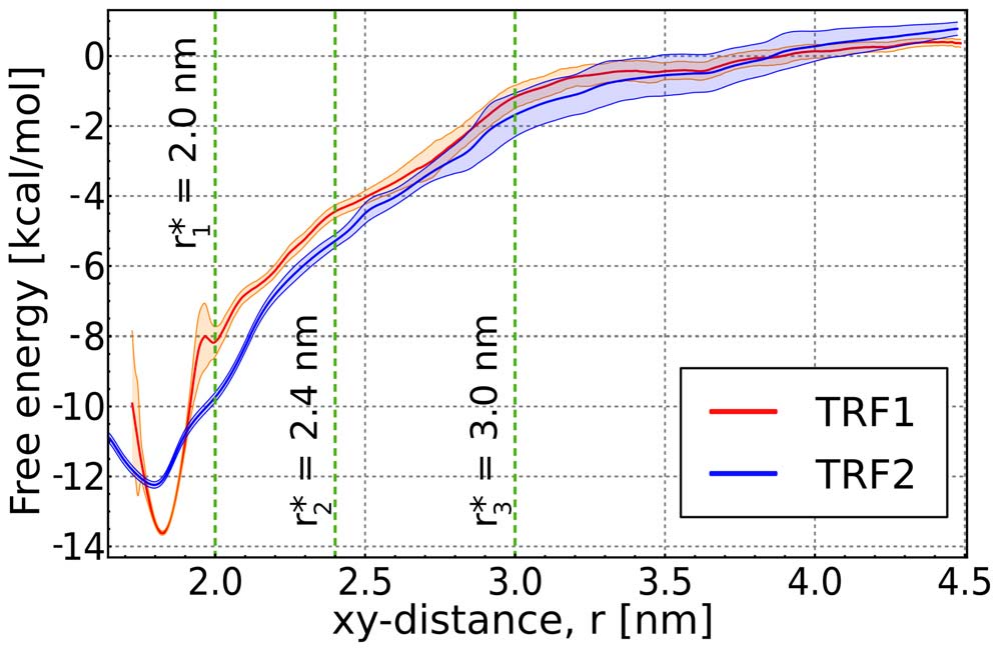
Free energy profiles for binding of TRF1 and TRF2 to telomeric DNA. Dashed green lines show the boundaries between the three defined bound states and the unbound state (for the corresponding binding free energies, see Table 1).

In an attempt to characterize and compare the affinity of TRF1 and TRF2 for telomeric DNA, we used the umbrella sampling (US) method to compute the free energy profiles for the binding of both proteins to the TTAGGG tandem repeat sequence (see Fig. 1A, Fig. S1 and Methods for details). The obtained profiles, shown in Fig. 2, share some common features, the most prominent of which is the overall shape of the free energy landscape that is dominated by electrostatic attraction between the positively charged proteins and the negatively charged DNA molecule. Another similarity is the absence of an activation barrier along the association pathway. This suggests that binding does not require major structural rearrangements of either the proteins or DNA, and the energetic cost of interface desolvation is insignificant in relation to the energy of association. Barrierless free energy profiles also indicate that the attractive interactions between the binding partners compensate for the entropic penalty associated with the loss of translational freedom (proportional to 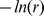) and thus may promote the kinetics of binding. Any possible loss of rotational freedom at intermediate xy-distances is also compensated by the electrostatic attraction (in fact, only slight deviations from the uniform distribution of the rotation angles are observed; see further discussion below). For both proteins, the minimum corresponding to a sequence-specific complex is located ∼1.8 nm from the DNA axis. The depth and width of this minimum are however substantially different: TRF1 binds to DNA more tightly and is therefore confined within a deep and narrow potential well, while TRF2 has a markedly broader and more shallow minimum region and can fluctuate more freely in the direction perpendicular to the DNA axis. This difference suggests that the precise nature of the binding mechanism might depend on the type of the protein.

Overall, our free energy profiles (Fig. 2) are in good agreement with the previous finding that TRF1 binds to the TTAGGG motif with higher affinity than TRF2. Indeed, in a recent experimental study by Hanaoka et al. [18] it was found that TRF1 and TRF2 differ in their affinity for telomeric DNA by 0.8 kcal/mol, with their absolute binding free energies equal to 9.2 and 8.4 kcal/mol, respectively. To directly compare our results with experimental data, we computed the respective free energies by integrating the probability distributions obtained from the free energy profiles as 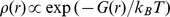, where 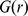 denotes the Gibbs free energy as a function of the xy-distance between the protein and the DNA axis, 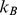 is the Boltzmann constant and 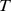 the temperature. The upper limit of integration for the unbound state was chosen so as to correspond to the standard concentration (see Methods for a detailed description of the procedure). The computed values of the binding free energies are summarized and compared with the experimental data in Table 1. As the quantitative discrimination between bound and unbound state can be to some extent arbitrary and method-dependent, a proper comparison between simulation and experimental data poses some difficulties. It is therefore crucial for investigation at the microscopic level that these states are defined reasonably. To more accurately examine the validity of our predictions, we calculated the binding free energies for three specific bound states (or binding modes), differing in the character of interaction and possibly reflecting consecutive stages of a protein/DNA association process. Our definition of the microscopic binding modes, depicted in Fig. S2, is further justified in the following sections by structural and mechanistic considerations based on the analysis of hydrogen bonding dynamics. The binding modes are defined by setting 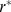, the boundary xy-distance between the binding partners that discriminates between the bound and unbound state. The most broadly defined bound state, the loose mode, with 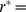 3.0 nm, encompasses also nonspecific complexes in which a contact between the binding partners is mediated by the N-terminal elastic region of the proteins. The intermediate mode (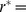 2.4 nm) starts from the initial contact between the C-terminal (recognition) helix and the DNA major groove, while the tight mode (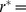 2.0 nm) corresponds to the sequence-specific binding in which the C-terminal helix interacts with the major groove bases.

**Table 1:** Standard binding free energies of the TRF DBD domains to telomeric DNA (kcal/mol).

	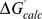			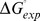 a
	tight,*r* ^*^ = 2.0^b^	intermediate,*r* ^*^ = 2.4	loose,*r* ^*^ = 3.0	
TRF1	−6.8	−9.8	−12.5	−9.2
TRF2	−4.3	−8.4	−11.5	−8.4

a experimental values taken from Ref. [18].

b *r* ^*^ (in nm) denotes a boundary between the bound and unbound state for the three defined binding modes.

Data in Table 1 show that for both proteins the absolute binding free energies corresponding to the intermediate mode give the closest agreement with the experimental values. This is probably due to the fact that the formation of a contact between the recognition helix and the DNA major groove is a process that best reflects the bound-unbound transition. In this interaction mode, most of the non-specific complexes between oppositely charged binding partners are left outside the bound state. Thus the obtained free energies are probably more compatible with the surface plasmon resonance results which are corrected for non-specific binding [18]. Since the umbrella sampling method has not been widely adopted to investigate DNA-protein interactions [22], [23], our results, showing that binding free energies can be reproduced to within a relatively small error, may be considered encouraging for future applications.

From Table 1 it can be also seen that, in the intermediate binding mode, a complex formed between telomeric DNA and TRF1 is by 1.4 kcal/mol more stable than in the case of TRF2. However, this free energy difference clearly increases with tightening the definition of the bound state, reaching its maximum value of 2.5 kcal/mol for a fully sequence-specific complex. This suggests that if in the experiment some of the less tightly-bound complexes are included into the bound population, the experimental free energies might actually underestimate the difference in binding-specificity between TRF1 and TRF2. The higher affinity of TRF1 for DNA, compared to its homologue, seems to arise from the overall and sequence-independent interactions rather than any differences in details of sequence recognition. Even though the latter exist, despite the highly conserved character of sequence-specific motifs (see Fig. 1 and further discussion), their influence on binding is thought to be of minor importance [18]. Molecular basis for this affinity difference is examined and discussed in the following sections.

### Determinants of Molecular Recognition in the TRF-DNA Complexes

It is important for understanding the mechanism of telomeric sequence recognition by TRF proteins to focus on specific interactions between protein residues and DNA bases. Interactions with the DNA backbone contribute to base sequence recognition only if the local DNA structure is strongly sequence-dependent, which is usually not the case. Specific interactions between amino acids and nucleobases are mediated by direct and water-bridged hydrogen bonds, with minor contribution from hydrophobic association [24], [25].

**Figure 3:**
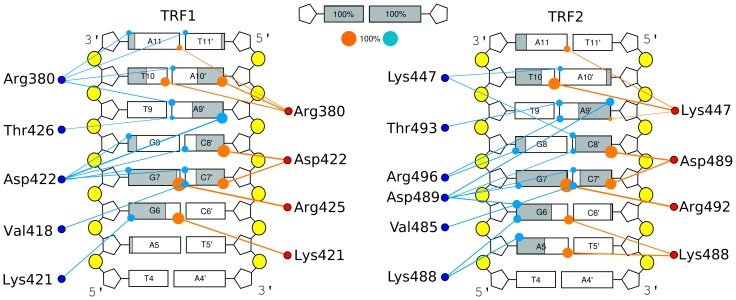
Direct and water-mediated hydrogen bonds between the TRF proteins and DNA bases. The pattern of direct and water-mediated hydrogen bonds between the TRF proteins and DNA bases. Only interactions between amino acid residues and nucleic bases are considered, as these base-specific contacts are potentially critical for sequence recognition. Filled circles at individual bases are scaled to reflect the probability that two residues are connected through a direct (orange) or water-mediated (cyan) hydrogen bond (for numeric probability values and hydrogen bond lifetimes, see Table S1 in File S1 and Table S5 in File S1). All contacts that are made with probability 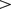
0.05 are included. Grey percentage bars show the probability for a given base to be involved, at any given time, in either direct or water-mediated hydrogen bonds with the protein.

1 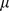
s equilibrium MD simulations performed for the tight binding mode show that the pattern of hydrogen bonds and water bridges between the TRF proteins and telomeric DNA is relatively dynamic, as depicted in Fig. 3 (for the full set of hydrogen bond data, including non-specific interactions with the DNA backbone, see Table S1–S8 in File S1). Not only do most water bridges exhibit moderate stability and often connect several DNA bases with a given amino acid, but also some of the direct contacts between the proteins and nucleobases are quite flexible. Although it may seem counterintuitive, this structural plasticity is thought to play an important role in DNA-protein recognition [26], as will be discussed below.

**Figure 4:**
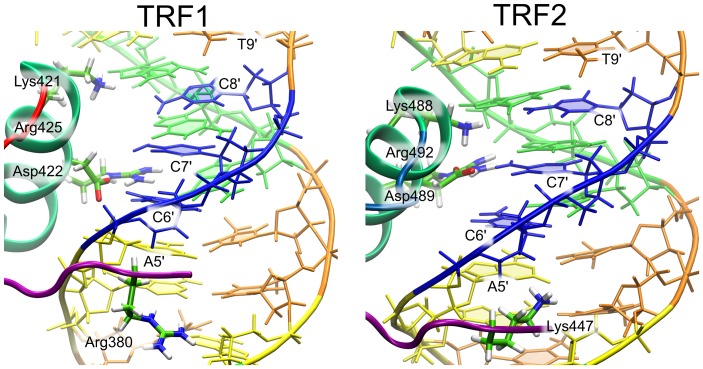
Detailed view of the interface between TRFs and DNA in the sequence-specific complex. Four amino acid residues important for recognition of telomeric dsDNA are shown explicitly. See legend to Fig. 1A for base color-coding.

It can be seen in Fig. 3 that for both TRF1 and TRF2, as few as four amino acid residues are responsible for the formation of virtually all of the sequence-specific hydrogen bonds. Three of these recognition sites (Lys-421/Lys-488, Asp-422/Asp-489, Arg-425/Arg-492 in TRF1/TRF2, respectively) are located in the C-terminal helix that binds in the major groove and one (Arg-380/Lys-447) accounts for the minor groove specificity of the N-terminal flexible region (Fig. 4). All these four residues are homologous (see Fig. 1D) which supports the assumption that the recognition mechanism is very similar for the two proteins (cf. similar pattern of dots in Fig. 3). The only significant difference in the hydrogen bonding pattern is a markedly higher hydration of the TRF2-DNA interface. This finding is in agreement with a less tight binding of TRF2 to DNA revealed by the free energy profiles.

**Figure 5:**
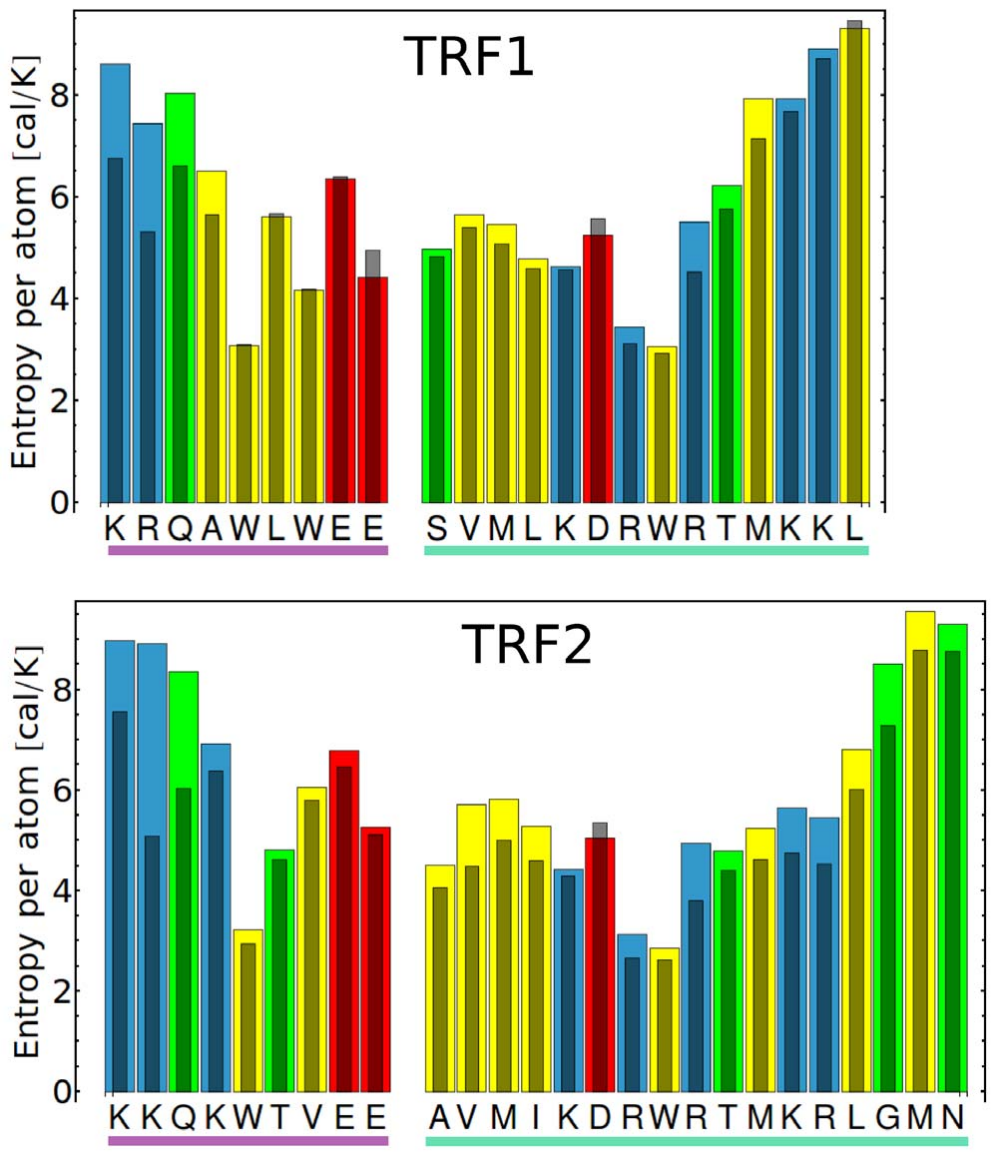
Protein entropy changes upon DNA binding. Conformational entropy per atom for individual protein residues in the tightly-bound complex with telomeric DNA (narrow bars) and in the isolated state (wide bars, colored according to amino acid type), estimated using the quasi-harmonic approximation. Only the regions interacting directly with DNA in the tightly-bound complex are presented: the N-terminal linker (purple) and the C-terminal helix (green).

**Figure 6:**
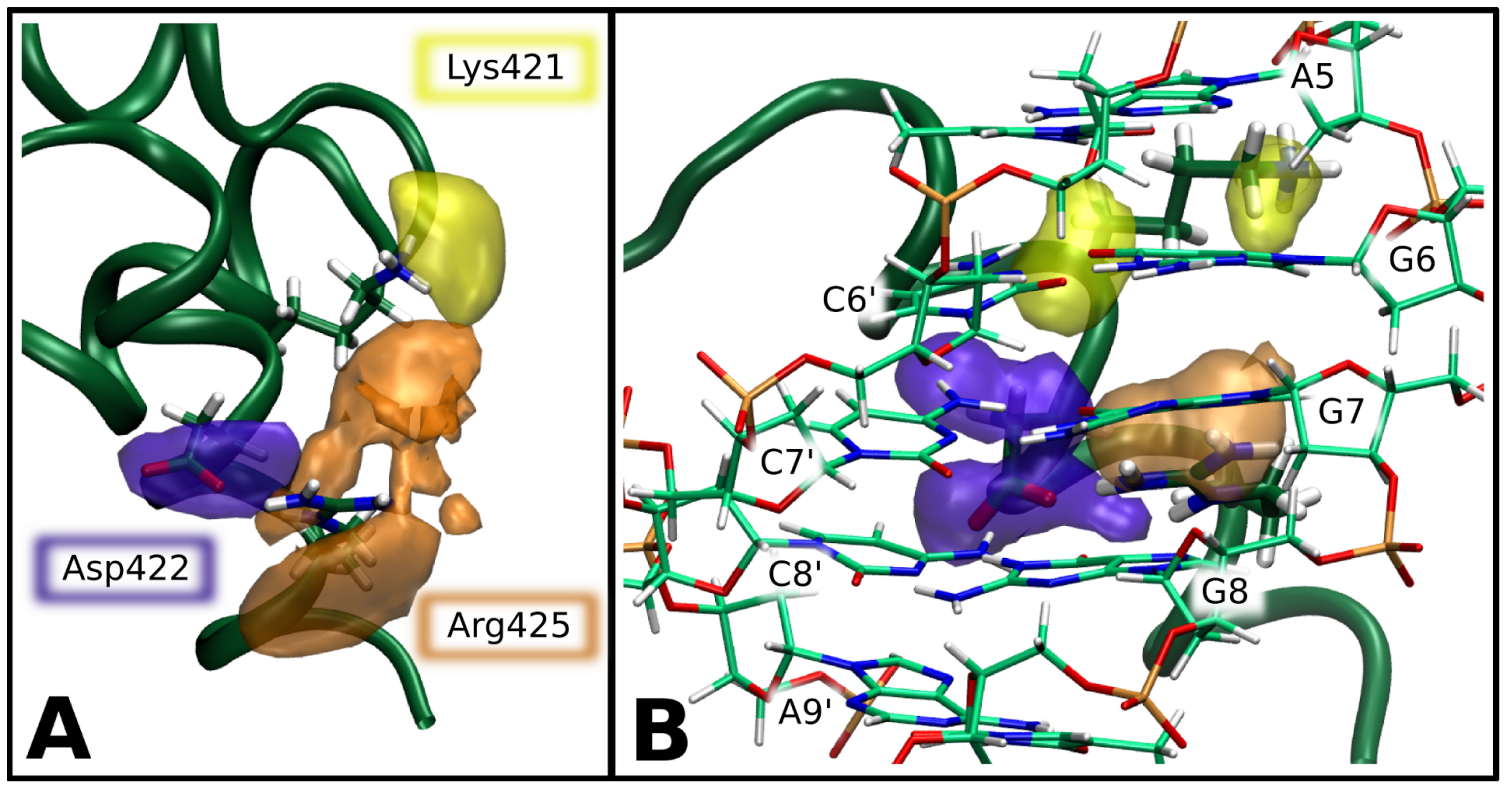
Spatial probability distributions for the critical residues of the TRF1 C-terminal helix. High-probability isosurfaces indicate conformations available to the recognition residues in the unbound (A) and tightly-bound (B) state. Distributions for Lys-421, Asp-422 and Arg-425 are plotted in yellow, blue and orange, respectively.

Since, on the one hand, an involvement in complex stabilization generally suppresses conformational fluctuations, but, on the other, the specific contacts at the TRF-DNA interface seem to be rather plastic, it was interesting to examine how the flexibility of individual protein residues changes upon DNA binding. To assess the effect of binding on the protein conformational fluctuations, we used the quasi-harmonic approximation to estimate the conformational entropy of each amino acid, separately for the bound and unbound state. As expected, the largest entropy changes were found for the protein regions directly interacting with DNA and are shown in Fig. 5. It can be seen that for most residues involved in binding, in particular these located in the flexible N-terminal region, the conformational entropy decreases upon binding. Surprisingly, however, a few side chains, especially negatively charged, are more flexible in the complex with DNA than in the unbound state. To better understand these unexpected results and to visualize the conformational dynamics of the DNA-recognition motif, in Fig. 6 we plotted the spatial distributions functions for the selected side chains in the C-terminal helix, at the interface with DNA and in the unbound state.

Taken together, the above findings led us to propose the following common mechanism of telomeric sequence recognition by TRF proteins. As it can be seen from Fig. 3 and Fig. 4, the basic side chain of Arg-425/Arg-492, located in the recognition helix, forms stable (ca. 90% probability) hydrogen bonds with a single base, the guanine residue G7, occasionally (ca. 10%) either replaced or further stabilized by a water-mediated interaction between the two residues. Apart from providing a specific interaction with G7, Arg-425/Arg-492 also forms a salt bridge with Asp-422/Asp-489, as seen in Fig. 4 and Fig. 6, therefore contributing substantially to the stability of this residue within the DNA-binding site. Our data suggest further that Asp-422/Asp-489 is a critical residue for specific recognition of the telomeric repeats by TRFs. First, the presence of an acidic residue within the binding site for a negatively charged DNA molecule might seem unexpected, especially if this energetically unfavorable location is evolutionarily conserved [27]. Another surprising result is that a significant increase in conformational entropy of Asp-422/Asp-489 (by 1.2–1.3 J/K per atom) is observed upon its binding to DNA, and this is the only such case in the recognition helix region (Fig. 5). This increase could be explained in terms of strong electrostatic repulsion between the negative charges, but an investigation of the spatial distribution of the side chains (Fig. 6) revealed another aspect of the interaction between Asp-422/Asp-489 and DNA. Within the major groove of telomeric DNA, Asp-422/Asp-489 switches between a number of conformations, seen as regions of high probability in Fig. 6B. By adopting these conformations the Asp side chain can interact favorably with five different sites at the DNA-protein interface: the amino groups of C7′ and C8′, the guanidinium group of the adjacent Arg residue and two water molecules, one forming a bridge with A9′ and another one easily exchanged with the bulk solvent. These favorable contacts, three of them being specific for the AGG sequence, effectively reduce the electrostatic repulsion between Asp-422/Asp-489 and DNA. Furthermore, the multiplicity of the stabilized conformations adopted by the Asp side chain at the interface partially reduces the entropic penalty associated with binding. Therefore, it can be expected that altering the DNA sequence would significantly increase the repulsion between the Asp-422/Asp-489 and DNA and restrict the conformational freedom of this amino acid, which effectively would lead to a decrease in the affinity of TRF for the altered DNA sequence. We propose that this negative-selection mechanism, based on minimization of unfavorable interaction with Asp-422/Asp-489, underlies the sequence specificity of TRF proteins. This, to our knowledge, is the first example of such a sequence recognition mechanism described in molecular detail. It can be thought of as a compensation effect, in which the overall DNA-binding affinity is lowered in order to ensure that the interaction is sufficiently sequence-specific. The overall affinity for DNA can then be restored, for example, by forming protein dimers and higher order oligomers, as it is the case for the TRF proteins and shelterin.

The importance of Asp-422/Asp-489 for the sequence recognition is also confirmed by our preliminary results on the effect of intercalation on TRF-DNA complex stability. We found that a specific triazoloacridone derivative (C-1305) [20] present between two adjacent GC pairs affects the local DNA structure and thus prevents Asp-422/Asp-489 from participating in favorable interactions at the interface. Consequently, a significant rise in the overall repulsion between the binding partners is observed (Fig. S3). To experimentally verify the proposed recognition mechanism, one could evaluate the effects of substitution of the critical Asp residue with Ala. According to our hypothesis, such mutation is expected to increase overall affinity for DNA, but at the same time disrupt the sequence-specific interactions and impair telomeric sequence recognition.

Our data indicate also that, compared to Arg-425/Arg-492 and Asp-422/Asp-489, the third key residue of the recognition helix, Lys-421/Lys-488, interacts with DNA less specifically (Fig. 3). In TRF1, Lys-421 relatively weakly (with ca. 60% probability) recognizes the G6 carbonyl group through both direct and water-mediated hydrogen bonds, while in TRF2, Lys-488 is likely to form a direct hydrogen bond with the A5 purine ring. It remains bound to A5 for half of the simulation time, while still “sensing” the G6 base through a relatively stable water bridge (almost 20%) and a direct hydrogen bond (30%). Considering both the competitive interaction with the adjacent phosphate group (as shown in Fig. 6, Table S4 in File S1 and Table S6 in File S1) and less specific contacts with nucleobases, we propose that Lys-421/Lys-488 plays only a secondary role in sequence recognition. This prediction can also be tested by mutation studies.

In order to validate our findings regarding the sequence recognition mechanism using phylogenetic data, we investigated the conservation of amino acid positions in the alignment of vertebrate Telomere Repeat-Binding Factors (binding to the TTAGGG repeat) and in the alignment of eukaryotic Telomere Repeat-Binding Proteins (binding to different telomere repeats). This analysis not only provides reference data for evaluating validity of our predictions, but also allows to correlate the recognition motif with the actual tandem repeat sequence. The results of the alignment, presented in Fig. S4 and Fig. S5, show that the KDXXR sequence in the C-terminal helix is highly conserved even among distantly related species, and suggest that this motif is specific for the T[A/T]GGG sequence in general. Therefore, the observed conservation pattern agrees very well with our molecular data indicating that Lys-421/Lys-488, Asp-422/Asp-489 and Arg-425/Arg-492 are involved in telomeric sequence recognition.

The role of Arg-380/Lys-447 as the minor groove binding residue (Fig. 4) seems to be insensitive to Arg/Lys substitution, as both of these positively charged residues seem to be interchangeable at this position in the TRF family sequence (Fig. S4). Nevertheless, these residues show different hydrogen bonding patterns in the DNA minor groove (Fig. 3). The guanidinium portion of arginine side chain is involved in the interaction with both T10 and A10′, while the lysine amino group interacts almost exclusively with T10 (Table S1 in File S1 and Table S5 in File S1).

High evolutionary conservation of three tryptophan residues, Trp-383/Trp-450, Trp-403/Trp-470 and Trp-424/Trp-491 (Fig. S4 and Fig. S5), might appear counterintuitive at first because these residues are not involved in sequence-specific interactions with DNA. Notably, there is only one codon for tryptophan therefore all Trp sites are non-degenerate. Consequently, the conservation of tryptophan residues must have been the result of purifying selection and the presence of tryptophan residues at these particular positions must be crucial for functionality of the domain. It was however suggested that the Trp residues play an important role in providing a hydrophobic scaffold crucial for maintenance of the DBD structure in the Myb proteins [28]. It is therefore likely that a similar explanation holds for other Myb-like domains, including TRF1 and TRF2. Our data indicate that, apart from their structural role, Trp-383/Trp-450 and, in particluar, Trp-403/Trp-470 can also participate in non-sequence-specific DNA binding by forming stable hydrogen bonds with phosphate groups (Table S2 in File S1 and Table S6 in File S1). Most other conserved positions can also be considered essential for the structural integrity of the domain. Among these residues, Glu-387/Glu-454 is involved in a salt bridge with Arg-415/Arg-482, Gly-399/Gly-466 and Gly-401/Gly-468 are located in the short turn between helices 1 and 2, and essentially all conserved residues with aliphatic side chains (i.e. Leu, Ile, Val) are buried in the hydrophobic core of the domain.

### Binding Mechanism of the TRF Proteins to Telomeric DNA

To provide insight into the mechanism of TRF binding to telomeric tandem repeats, we investigated how major contributors to the binding free energy, including hydrogen bonds, interaction energies and solvent-accessible surface areas, depend on the xy-distance between the protein and DNA. These profiles, allowing a detailed analysis of the overall binding energetics, were obtained by evaluating equilibrium ensemble averages over the umbrella sampling trajectories, using a weighting factor of 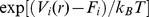, where 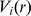 and 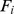 are the US biasing potential and the WHAM free energy constant, respectively, both corresponding to the 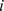-th US window.

**Figure 7:**
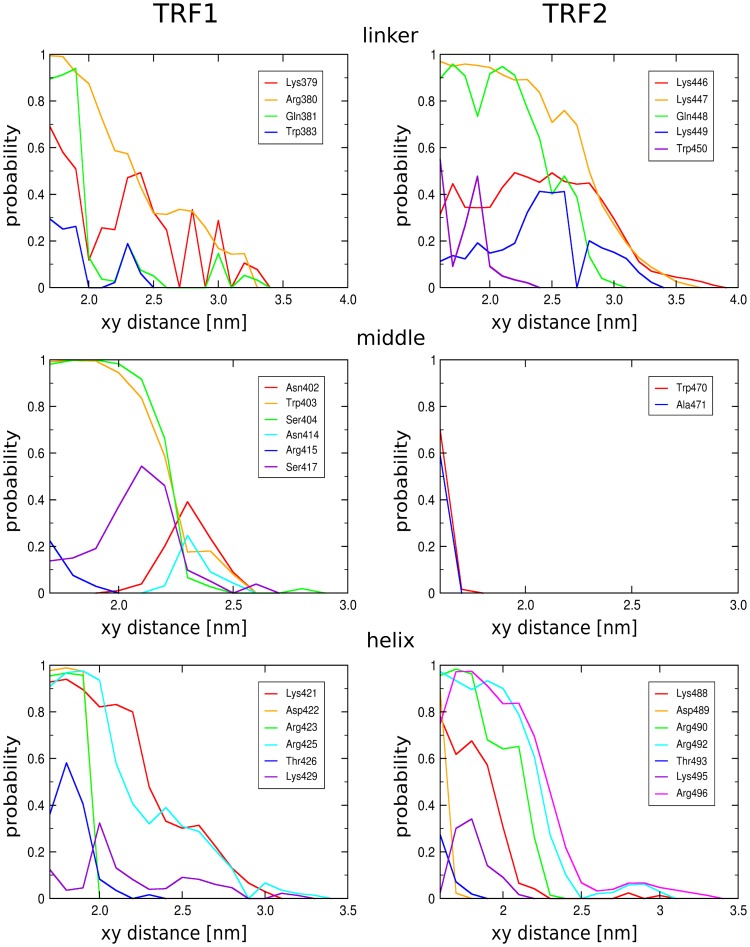
Hydrogen bond profiles for individual TRF residues. The probability of hydrogen bond formation between individual protein residues and DNA as a function of the distance from the DNA axis. Only the residues for which the probability exceeds 0.2 are presented. The protein structure is subdivided into three separate regions: the N-terminal linker (top), the middle region (middle) and the C-terminal helix (bottom).

The probabilities of forming hydrogen bonds between the different protein regions and DNA, shown in Fig. 7 as a function of the xy-distance between the binding partners, shed light on the strength and range of the complex-stabilizing interactions and therefore allow to speculate on the precise mechanism of the association process. It can be seen from Fig. 7 (bottom panel) that for the critical residues in the C-terminal helix of TRF1 the probability of forming hydrogen bonds with DNA is high in the tightly-bound state and decreases rapidly with increasing distance. This abrupt change in DNA-protein interactions, especially evident for Asp-422, Arg-423 and Arg-425, gives rise to a narrow and fairly deep well in the free energy profile for to the TRF1-DNA complex (Fig. 2). In contrast, the C-terminal helix of TRF2 interacts favorably with DNA over somewhat longer distances from the duplex axis, allowing for greater flexibility of the TRF2-DNA complex. This difference is even more pronounced for the N-terminal positively charged linker which at larger distances is found to protrude from the protein to make contact with the DNA minor groove (Fig. 7, top panel). Despite these differences, the probability profiles in Fig. 7 confirm the above finding that the pattern of sequence-specific hydrogen bonds is similar for TRF1 and TRF2.

For both proteins, the probability of forming hydrogen bonds between the recognition helix and DNA falls off steeply as the xy-distance exceeds 2.4 nm. This marked feature was used to define the threshold distance, 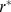, for the above described intermediate binding mode. Beyond this distance, there is very little contact between the helix and DNA, mostly mediated through long side-chain residues. On the contrary, the N-terminal linker, especially in the case of TRF2, can form stable hydrogen bonds with DNA even at distances up to 3.0 nm, and therefore this distance was used as a boundary for the loose mode. The linker appears to be strongly attracted to the minor groove and the DNA backbone, and provides a means of anchoring the protein to the DNA duplex in the loosely-bound state. At intermediate distances, it dynamically binds to and unbinds from the DNA backbone, as can be seen in Fig. S6. This indicates that the observed long-range interactions are not an artifact of the non-equilibrium pulling simulations and therefore additionally supports the idea that the linker region may be involved in anchoring the protein to the DNA duplex. It is worth noting here that the simulated DNA-binding domain encompasses only a short C-terminal fragment of the flexible linker. In the entire protein, this fragment is preceded by a region rich in positively charged residues that may additionally contribute to this non-specific binding effect. It has been shown [29] that Thr phosphorylation in this region disfavors the binding of TRF1 to DNA, possibly via either electrostatic or structural changes, suggesting an important yet unknown role of the preceding linker fragment in the anchoring effect.

This observation supports the model of the binding equilibrium in which the total population of DNA-bound TRF consists of two structurally distinct subpopulations: loosely-bound molecules, attached to DNA mainly through the N-terminal linker, and tightly-bound molecules, with the C-terminal helix interacting with the AGGG motif in the major groove. Within this model, the tightly-bound proteins are necessarily confined to the recognition site, whereas in the loosely-bound state they may diffuse relatively freely along the DNA strand while maintaining contact with DNA via the extended N-terminal linker. Thus this picture of the binding equilibrium favors 1D-sliding as a mechanism of accelerated sequence searching. In this mechanism a non-specifically bound protein searches for its target sequence by making a random walk along the DNA strand and binds tightly only when the target site is found [30]. Certain DNA-binding proteins have been shown to utilize this mechanism to overcome the 3D diffusion limit and accelerate the search for a specific target site on DNA [31]. The validity of this binding mechanism depends, though, on a higher-dimensional free energy landscape and in the case of TRF proteins should be subject to further research.

Fig. 7 also clearly shows that there are significant differences in the pattern of hydrogen bonds formed between the “middle” region of both proteins and the DNA molecule. In the case of TRF2, virtually all interactions between this region and DNA vanish beyond 1.7 nm, while TRF1 is strongly bound through its Trp-403, Ser-404 and Ser-417 to the DNA phosphate backbone (see Table S2 in File S1) up to the intermediate mode boundary at 2.3–2.4 nm. This difference is consistent the with experimental results on the substitution of Ser-404 and Ser-417 by alanines, which occur at these positions in TRF2 [18]. Stronger interactions observed for the middle region of TRF1 should also facilitate proper orientation of the recognition helix with respect to the major groove and thereby cooperatively promote tighter binding of this protein. In general, the differences found for the middle region could partially explain the higher affinity of TRF1 for telomeric DNA and support the previous results suggesting a non-specific nature of the binding free energy difference.

**Figure 8:**
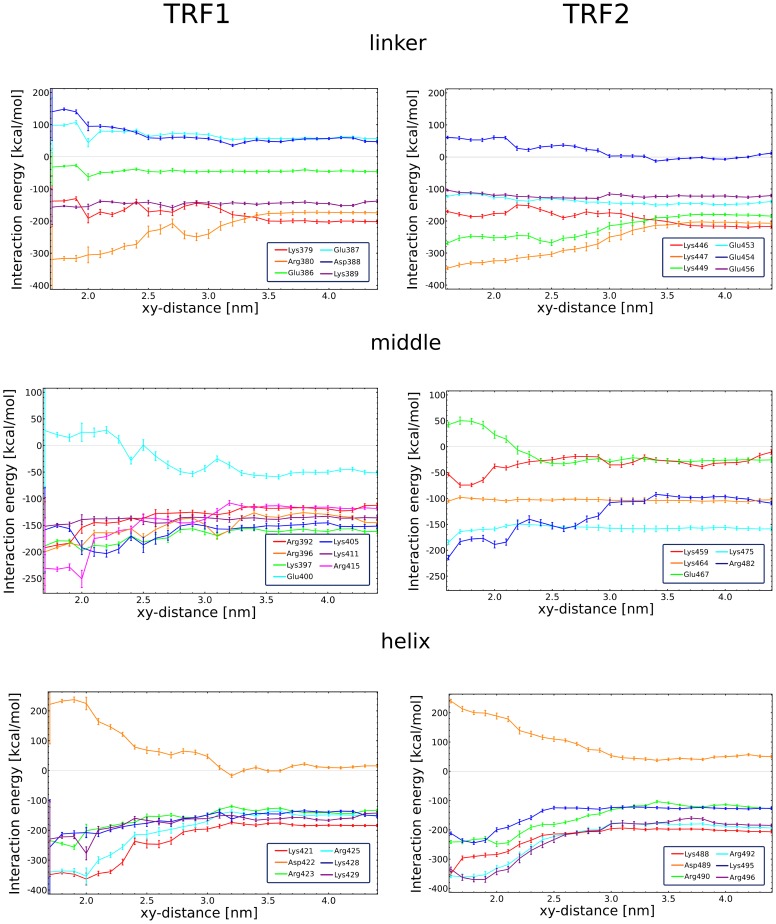
Interaction energy profiles for individual protein residues. Interaction energy (computed as a sum of electrostatic and van der Waals contributions) between the charged amino acid residues and their surroundings (DNA and solvent combined) as a function of the distance between the protein and the DNA axis. The protein structure is subdivided into three separate regions: the N-terminal linker (top), the middle region (middle) and the C-terminal helix (bottom). For a full set of amino acid interaction energies, see Fig. S7.

A similar conclusion can be drawn from Fig. 8, showing how the overall interaction energies of the charged amino acids with their surroundings (DNA and solvent) change with the xy-distance between the complex constituents (see also Fig. S7). Not only does the middle region of TRF1 interact more readily by means of direct hydrogen bonds with DNA, but also, as can be seen in Fig. 8, contains more positively charged residues, strongly attracted to the DNA phosphate backbone. This finding strongly suggests that the difference in the DNA-binding affinity observed between TRF1 and TRF2 is of enthalpic origin and results from the sequence-nonspecific interactions with the DNA backbone. Interestingly, the positively charged residues in the N-terminal linker region (Fig. 8, top panel) seem to partially counteract this effect, as in the case of the TRF2-DNA complex they are found to provide more stabilization. This shift of the stabilizing effect towards the linker is consistent with and may explain the previous observation that the binding free energy difference decreases when the bound state is defined to include less tightly bound complexes (see Table 1). In the case of TRF1, the attraction mediated by the middle region of the protein makes the complex more compact and facilitates tight binding by the additional stabilization of the recognition helix in the major groove. In contrast, the higher affinity of the linker for the minor groove and the DNA backbone, observed for TRF2, does not exert a tightening effect but stabilizes the loose, non-sequence-specific complexes, effectively reducing the binding free energy difference for the loose mode, as defined in Table 1. The differences in the interaction energy profiles found between the C-terminal helix of both proteins (Fig. 8, bottom panel) are much less pronounced than for the two remaining regions.

The observed interaction energies also explain why there is no enthalpic barrier in the free energies profiles (Fig. 2) for the intermediate DNA-protein separations at which the partial desolvation of the approaching surfaces could not yet be compensated by the electrostatic attraction between the binding partners. Indeed, Fig. 8 and Fig. S7 indicate that, with a few individual exceptions (e.g., Arg-380 in TRF1, Arg-482 in TRF2), the overall attraction between the protein and DNA dominates the energetics of the binding process over the entire separation range and easily compensates for the energetic cost of surface desolvation.

**Figure 9:**
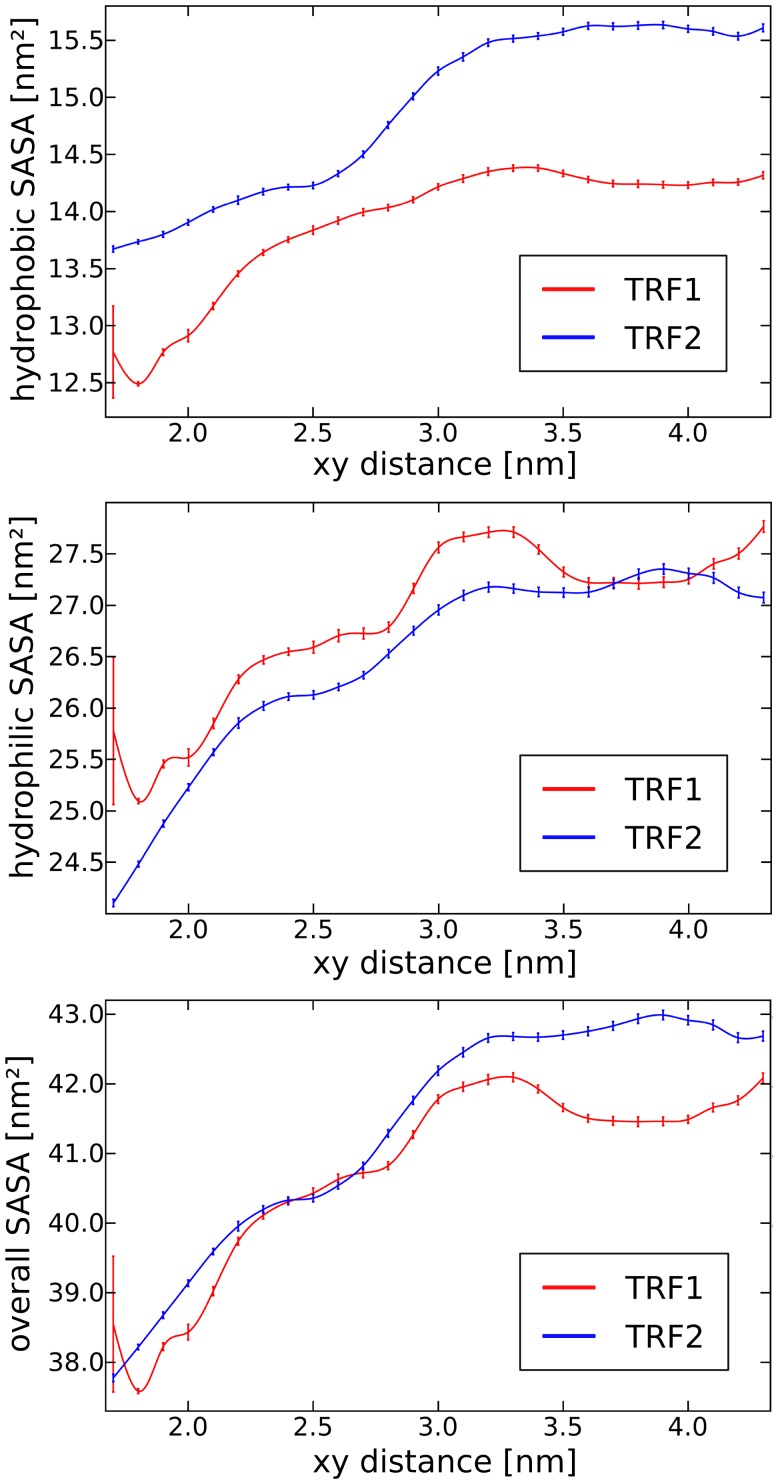
Solvent accessible surface of TRFs as a function of their distance from the DNA axis. Hydrophobic (top), hydrophilic (middle) and overall (bottom) solvent accessible surface area of TRF1 and TRF2 as a function of the xy-distance between the protein and the DNA dodecamer.

As hydrophobic association might be also involved in the process of protein binding to DNA, we investigated the dependence of the solvent accessible surface area (SASA) on the separation between the complex constituents. The obtained profiles, shown in Fig. 9, reveal that the SASA of TRF2 that is buried upon DNA binding is larger by ca. 1 nm^2^ compared to TRF1. Since at the room temperature, an increase in the solvent entropy is approximately proportional to the loss of SASA at the protein-DNA interface, it can be argued that this entropic contribution to the binding free energy is more favorable for TRF2 than for TRF1. This result, if valid, would be consistent with the previous conclusions that the higher stability of TRF1-DNA complexes is of enthalpic origin.

Although the SASA profiles obtained for TRF1 and TRF2 share many common features, it can be seen from the top panel of Fig. 9 that the largest change in the hydrophobic SASA occurs between 1.9 and 2.5 nm for TRF1 and between 2.5 and 3.1 nm for TRF2. Decomposition of SASA into per-residue contributions (Fig. S8) shows that these changes are due to a few basic residues involved in the complex stabilization: Lys-421, Arg-380 and, to some extent, Lys-429 in TRF1 and Lys-447 with a small contribution from Lys-449 in TRF2. Indeed, the observed increase in the hydrophobic SASA upon dissociation results from the exposure of the aliphatic chains of the above basic residues whose positively charged hydrophilic groups are attached to the backbone through 4 (Lys) or 3 (Arg) hydrophobic methylene groups. These hydrophobic parts are buried in the complex interface in the bound state and become exposed to the solvent as the distance between the binding partners increases. Because in this case the entropic penalty cannot be compensated by the interactions with the solvent, such exposure is particularly unfavorable. Thus, owing to their amphiphilic nature, the basic amino acids might stabilize the TRF-DNA complex by both direct interaction with the polar groups of DNA and hydrophobically driven association mediated by their aliphatic fragments. As most of the residues involved in the hydrophobic SASA change belong to the N-terminal linker, the stronger interaction between this region and the minor groove observed for TRF2-DNA might explain the shift seen in the top panel of Fig. 9.

**Figure 10:**
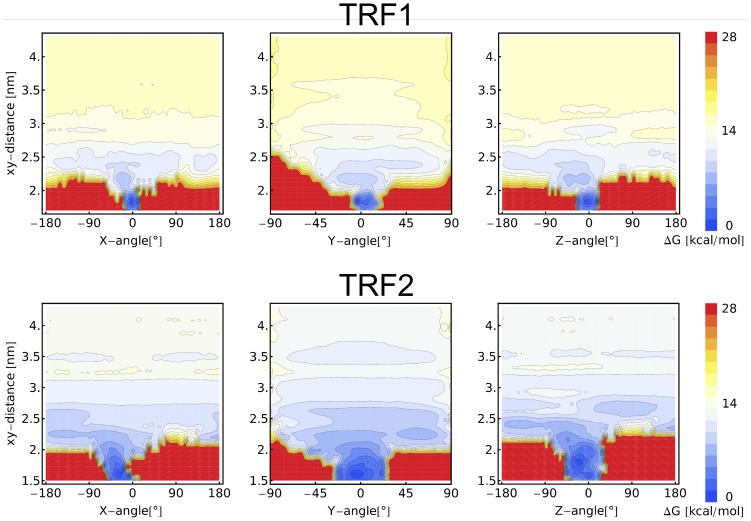
Orientations of TRF1/TRF2 DBDs with respect to DNA as a function of xy-distance. Two-dimensional free energy surfaces for the xy-distance and the three rigid-body rotation angles defining the orientation of TRF1 and TRF2 DBD domains with respect to the DNA dodecamer (aligned with the z axis). To calculate the angles, we first obtained a rotation matrix which gives the best-fit of the instantaneous protein structure to the initial one in the tightly-bound complex with DNA. The Rotation matrix was then expressed as a product of three matrices representing rotations about the x, y and z axis and the rotation angles were derived from these matrices.

It is notable that the DBD domains of TRF proteins exhibit a certain degree of charge polarization, i.e., the regions facing DNA in the tightly-bound complex are more positively charged than these exposed to the solvent at the opposite side (see in Fig. 1). Therefore, it could be expected that the negatively charged DNA strand would help orient the protein molecule in a way that would accelerate the binding kinetics. To determine if such pre-orientation does occur, we computed the 2D free energy surfaces for xy-distance and the three rigid-body rotation angles by Boltzmann-inverting the respective joint probability distributions, obtained by proper reweighting of the umbrella sampling data. (Fig. 10). It turned out that beyond the distance at which the recognition helix starts to contact the major groove (2.4 nm), very little pre-orientation is observed, as indicated by almost uniform distributions in Fig. 10. One can however argue that up to 3.0 nm, i.e, as long as the linker contacts the minor groove, there is a certain bias in the distribution of the rotation angle around the z axis. Furthermore, Fig. 10 confirms the difference in the flexibility of binding between TRF1 and TRF2. From the obtained free energy surfaces it is clear that TRF2 binds to DNA less tightly as in the bound state its rotational fluctuations are approximately twice as large as those seen for TRF1. In addition, the uniform distribution of the rotation angles at large distances can be considered indicative of adequate sampling of protein orientations with respect to the DNA duplex.

## Conclusions

In this work, molecular recognition in the complexes of the TRF1 and TRF2 proteins with telomeric DNA was studied by computing the corresponding free energy profiles. The binding free energies derived from these profiles seems to be in good agreement with the experimental data [18] and the higher affinity of TRF1 for DNA is also reproduced in the calculations. Further, the main contributions to the overall free energy profiles were thoroughly analyzed to elucidate the molecular determinants of sequence specificity in the the TRF-DNA interactions. In particular, by examining the pattern of direct and water-mediated hydrogen bonds we identified the TRF residues involved in recognition of double-stranded telomeric DNA. Binding enthalpies and entropies determined for these residues provided valuable insight into their role in TRF-DNA complex formation. These findings were further tested using phylogenetic data in order to reinforce our conclusions. Finally, we also examined the mechanism of the TRF-DNA binding by investigating how major contributors to the binding free energy vary with the distance between the protein and DNA.

Our results suggest that the affinity difference between TRF1 and TRF2 is not directly related to the mechanism by which the TRF proteins recognize telomeric DNA. We propose that for both proteins this mechanism is the same and is based largely on a decreased electrostatic repulsion between a single aspartate residue in the C-terminal (recognition) helix and DNA. The negatively charged Asp-422/Asp-489 (in TRF1/TRF2, respectively), normally repelled by the negative charges on the DNA phosphate backbone, at the target site is involved in several attracting interactions with DNA bases (AGG motif), which effectively reduce the repulsion. The ability of Asp-422/Asp-489 to adopt multiple energetically stabilized conformations at the target site decreases the entropic penalty associated with binding and additionally enhances the “negative selection” effect. At the DNA/protein interface, Asp-422/Asp-489 is stabilized by Arg-425/Arg-492, which by itself contributes to sequence recognition by forming a specific hydrogen-bond contact with a single guanine base. The involvement of these two residues in sequence recognition is further supported by evolutionary conservation patterns. However, further research is necessary to quantitatively evaluate their precise role in sequence-specific binding. For instance, the D422A and D489A mutants of TRF1 and TRF2, respectively, could be used to test our predictions, as according to the proposed mechanism they should bind DNA even more strongly than the wild-type forms, though at the cost of decreased sequence specificity. Other TRF residues at the DNA/protein interface are characterized by weaker and less specific interactions with DNA bases, and are therefore expected to play a minor, supportive role in targeting the telomeric repeat sequence.

The obtained free energy profiles also indicated the differences in the interactions of TRF1 and TRF2 with DNA. Importantly, we found that TRF1 binds to the telomeric sequence more tightly, within a deep and narrow free energy well, whilst TRF2 forms a more flexible complex, with a substantially broader and more shallow minimum region. At the microscopic level, this difference may be explained by the stronger electrostatic interactions and hydrogen bonding between the DNA backbone and the middle region of TRF1 (the region between the N-terminal linker and C-terminal recognition helix). These non-sequence-specific interactions mediated through the protein middle region cooperatively promote tighter binding of the C-terminal helix to the major groove and seem to be responsible for the higher affinity of TRF1 for telomeric DNA.

We also propose a simple model of the TRF/DNA binding equilibrium in which DNA-bound proteins bind to the double helix either tightly, via the C-terminal helix involved in sequence-specific interactions in the major groove, or loosely, through the N-terminal linker maintaining a non-specific contact with the minor groove or the DNA backbone. This model provides a conceptual framework for understanding the mechanism of TRF-DNA interactions and suggests that the TRF proteins may search for their target sequence by performing a 1D random walk along the DNA strand, with an extended, positively charged N-terminal linker possibly acting as a flexible holding “arm”.

## Methods

### Molecular Systems

Initial coordinates of the TRF-DNA complexes were taken from the 2.0-Å and 1.8-Å resolution crystal structures containing two TRF1 and two TRF2 DNA-binding domains, respectively, bound to telomeric dsDNA (Protein Data Bank IDs: 1W0T and 1W0U [19]). These structures were processed to obtain single DBD domains bound in the middle of a 12-bp DNA duplex (dodecamer) with the sequence 5′-GGTTAGGGTTAG-3′ (Fig. 1). Protonation states were assigned using the Propka program [32]. The N-termini of both DBD domains were acetylated, since these are not the native N-terminal ends of a TRF molecule. The systems were solvated with TIP3P water and neutralized by adding K^+^ and Cl^−^ to obtain the physiological ionic strength of 0.15 M. K^+^ was chosen as a counterion to mimic high intracellular potassium concentration. The equilibrium MD simulations of the TRF1-DNA and TRF2-DNA complexes and the isolated TRF1 and TRF2 DBD domains were performed in rhombic dodecahedron boxes with box vectors of 7.201, 7.204, 7.260 and 7.265 nm and 8108, 8104, 8529 and 8398 water molecules, in respective order. The umbrella sampling simulations were carried out in cubic boxes with vector lengths of 7.344 and 7.425 nm, containing 12376 and 13692 water molecules for TRF1-DNA and TRF2-DNA, respectively.

### Simulation Procedure

All molecular dynamics (MD) simulations were performed using Gromacs 4.5.5 package [33] and AMBER99SB force field [34] with the PARMBSC0 refinement [35]. Periodic boundary conditions were applied in all three dimensions. The electrostatic interactions were calculated using the particle-mesh Ewald method [36], with Fast Fourier Transform grid spacing of 0.12 nm. Coulombic real space and Lennard-Jones interactions were truncated at 1 nm. The Berendsen weak coupling algorithm [37] was used for temperature and pressure control, with reference values of 300 K and 1 bar and time constants of 0.1 ps and 2 ps, respectively. The water molecule geometry was constrained using SETTLE [38] and the P-LINCS [39] method was used to constrain all the remaining bond lengths. The equations of motion were integrated using the leap-frog integration algorithm with a timestep of 2 fs. All equilibrium MD simulations of the TRF-DNA complexes and the isolated TRF proteins were performed for 1 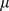
s each.

### Free Energy Calculations

The free energy profiles for binding of the TRF proteins to the telomeric DNA duplex were calculated using the umbrella sampling procedure [40]. In this method, a biasing potential is applied to the system in order to enhance sampling of low-probability configurations along a defined reaction coordinate. In our case, the reaction coordinate was chosen to be the xy-plane projection of the distance between the centers of mass (COM) of the protein and the DNA dodecamer (xy-distance), while the latter was rotationally restrained to maintain its orientation along the z axis. To avoid the effects of DNA bending fluctuations on the observed xy-distance distributions, the position of the DNA COM was defined as the center of mass of all heavy atoms of the four central DNA base pairs (5′-AGGG-3′) which, in a tightly-bound complex with TRF, are in direct contact with the protein (for a schematic illustration of the chosen reaction coordinate, see Fig. S1). In our setup, the reaction coordinate represents the actual distance between the protein and the DNA axis and thus allows for a clear distinction between the bound and unbound state. At the same time, the protein motions along the DNA strand (z axis) are not restricted in order to allow for a full description of the binding equilibrium including non-sequence-specific and less-tightly bound complexes.

Initial coordinates for the sampling procedure were obtained from the steered MD simulations in which the protein was separated from DNA during a 100 ns long run. Sixteen umbrella sampling windows were used, with a harmonic biasing potential (force constant of 700 kJ mol^−1^nm^−1^) centered on successive values of xy-distance from 1.5 to 4.5 nm with a 0.2 nm step. The systems were simulated for 1 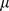
s in each umbrella window using the same MD protocol as described above. The WHAM procedure [41] was used to determine the free energies as a function of the reaction coordinate. For standard error calculations, autocorrelations in the sampled time series were taken into account.

To approximate the standard binding free energies of TRF proteins to telomeric DNA, the probability distributions derived from the free energy profiles, 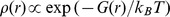, were integrated over the bound (*b*) and unbound (*u*) states:
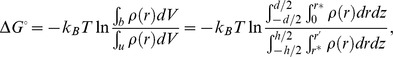
where 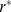 is an arbitrarily chosen boundary between the bound and unbound state in the radial direction. The upper integration limit for the unbound state, 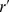, is chosen so as to correspond to the standard concentration 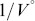, i.e., it is obtained from the equation 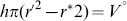, where 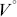 is 1.661 nm^3^. The integration interval along the *z* direction, *d*, denotes the range of protein spatial fluctuations along the DNA axis in the bound state and *h* is the length of the simulation box in the *z* direction. It should be noted, however, that the obtained free energy differences depend only very weakly on the particular choice of *h*, as they are dominated by the bound state probability, i.e., by the choice of 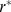. Note also that the probability distribution function 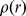 is obtained directly from the original free energy profiles and as such it approximates marginal probability density, with the angular coordinate (the polar angle) integrated out.

### Phylogenetic Analysis

Protein sequences of Telomeric Repeat-Binding Protein family (PTHR21717) [42], containing human TRF1 and TRF2, were downloaded from UniProt database [43]. The full sequence alignment was performed using Clustal Omega [44] and conserved blocks were chosen for further analysis. Phylogenetic tree was calculated using Maximum Likelihood method with GAMMA model of rate heterogeneity and WAG Amino Acid substitution model using RAxML [45].

## Supporting Information

Figure S1 **Definition of the reaction coordinate used in the umbrella sampling simulations.** With DNA restrained so as to maintain its main axis parallel to the z-axis, the coordinate is described as an xy-projection of the vector connecting four DNA base pairs (5′-GGGT-3′) and the protein molecule. Centers of mass are used as the reference points for these two groups of atoms.
(TIFF)

Figure S2 **Representative structures of the three defined binding modes.** The tight mode (up to 2.0 nm) corresponds to the sequence-specific protein-DNA complex, where amino acid residues of the major groove-binding helix (shown in turquoise) can form hydrogen bonds with the DNA bases. In the intermediate mode (up to 2.4 nm), the helix contacts the DNA also through non-specific interactions. The loose mode (up to 3.0 nm) encompasses also the states where the contact is only maintained through the N-terminal linker. Each state is visualized in three projections – on the XZ (side), YZ (front) and XY (top) plane.
(TIFF)

Figure S3 **Changes in interaction energies between individual residues of TRF1 and its surroundings (DNA and solvent combined) upon C1305 intercalation.** Wide bars, colored according to amino acid type, show interaction energies in the absence of C-1305, while grey narrow bars correspond to respective energies after intercalation. In the simulation, the interacalating compound is inserted between the first and second GC pair in a GGG motif. Only the N-terminal linker and C-terminal helix regions are shown (cf. Fig. 5 in the main text). Note that strong repulsion of the rightmost Leu residue is due to its negatively charged C-terminal carboxyl group.
(TIFF)

Figure S4 **Evolution of Telomere-Repeat Binding Factors in vertebrates.** Phylogenetic tree of vertabrate Telomeric Repeat Binding Factors calculated with RAxML and the alignment of DNA repet binding region with conserved amino acid positions marked in blue.
(TIFF)

Figure S5 **Evolution of Telomere-Repeat Binding Proteins in vertebrates.** Phylogenetic tree of eukaryotic Telomeric Repeat Binding Proteins calculated with RAxML and the alignment of DNA repeat binding region with conserved amino acid positions marked in blue. On the right, colored dots correspond to different target sequences of the DNA-binding domains.
(TIFF)

Figure S6 **Dynamics of the flexible linker.** Time series plots of xy-distance between the DNA and the linker for intermediate sampling windows (corresponding to reaction coordinate values from 2.2 to 3.0 nm) are shown to highlight sufficient sampling of the linker conformations in the study. The black line, corresponding to the tight complex, is shown for reference. In the plots, multiple binding and unbinding events can be seen, with noticeable baselines at ca. 0.5 nm (interactions with the minor groove) and ca. 1 nm (interactions with the DNA backbone). For distance calculation, a terminal side chain heteroatom of the respective residue was used as a reference group.
(TIFF)

Figure S7 **Interaction energy profiles for individual protein residues.** Interaction energy (computed as a sum of electrostatic and van der Waals contributions) between all amino acid residues of TRF1 (top) and TRF2 (bottom) and their surroundings (DNA and solvent combined) as a function of the distance between the protein and the DNA axis.
(TIFF)

Figure S8 **Hydrophobic solvent accessible surface area of TRF1 and TRF2 as a function of their distance from the DNA axis.** The observed increase in the hydrophobic SASA upon dissociation (top) results mainly from the exposure of the three basic residues in TRF1 and one basic residue in TRF2 (bottom).
(TIFF)

File S1 **Tables S1–S8 present statistical data on direct and water-mediated hydrogen bonds between the TRF proteins and DNA.** The results are sorted by protein (TRF1/TRF2), type of interaction (direct or water mediated hydrogen bond) and sequence-specificity (interaction with DNA bases or backbone). DNA base pairs are numbered consecutively as in the sequence 5′-GGTTAGGGTTAG-3′, with a prime symbol (’) indicating numeration of the complementary strand.
(PDF)
