# Supplementary figures and images for: Molecular Recognition in Complexes of TRF Proteins with Telomeric DNA
Source: PLoS One. 2014 Feb 26;9(2):e89460. doi: 10.1371/journal.pone.0089460 (PMC3935891; doi:10.1371/journal.pone.0089460)

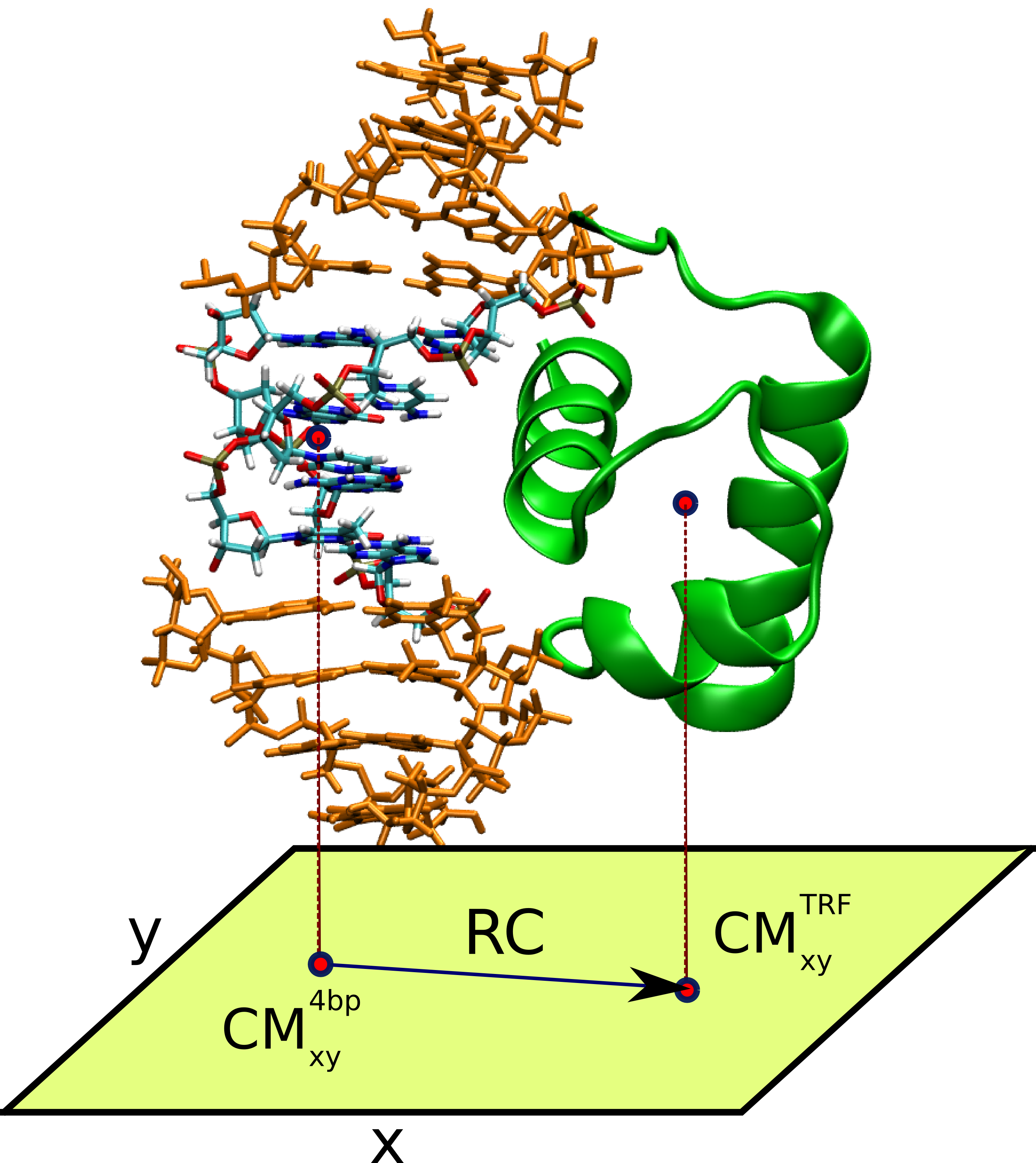

Supplement: Figure S1 — Definition of the reaction coordinate used in the umbrella sampling simulations. With DNA restrained so as to maintain its main axis parallel to the z-axis, the coordinate is described as an xy-projection of the vector connecting four DNA base pairs (5′-GGGT-3′) and the protein molecule. Centers of mass are used as the reference points for these two groups of atoms. (TIFF) [file pone.0089460.s001.tiff]

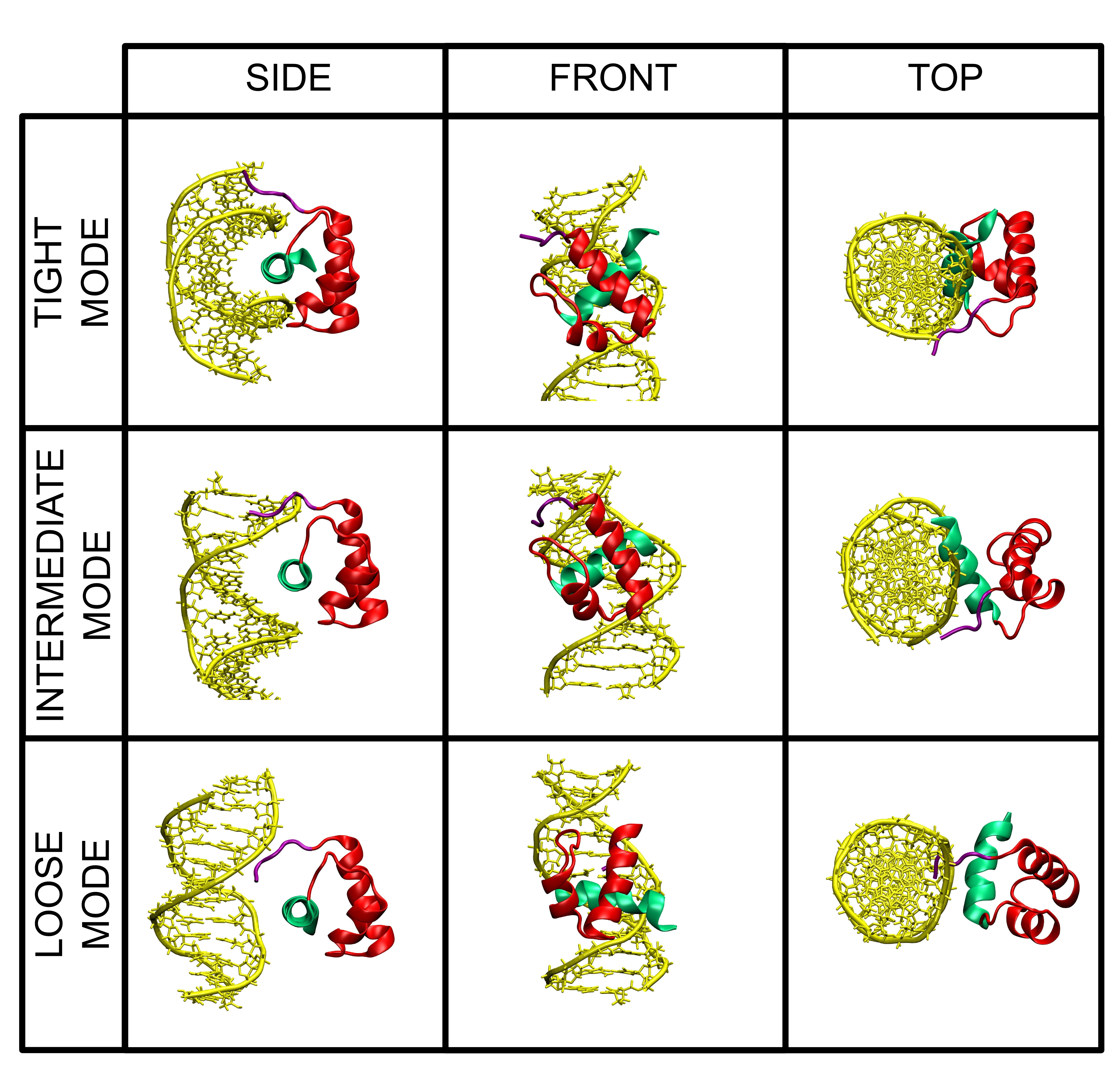

Supplement: Figure S2 — Representative structures of the three defined binding modes. The tight mode (up to 2.0 nm) corresponds to the sequence-specific protein-DNA complex, where amino acid residues of the major groove-binding helix (shown in turquoise) can form hydrogen bonds with the DNA bases. In the intermediate mode (up to 2.4 nm), the helix contacts the DNA also through non-specific interactions. The loose mode (up to 3.0 nm) encompasses also the states where the contact is only maintained through the N-terminal linker. Each state is visualized in three projections – on the XZ (side), YZ (front) and XY (top) plane. (TIFF) [file pone.0089460.s002.tiff]

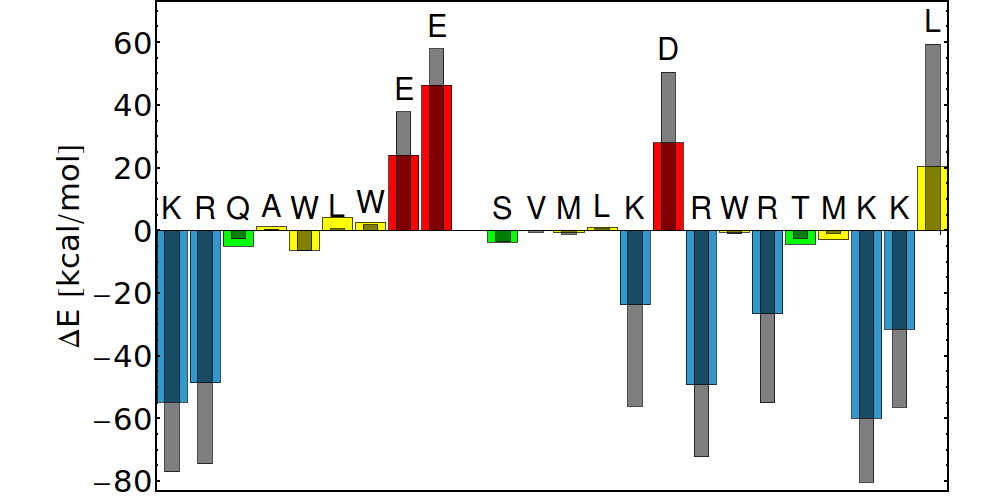

Supplement: Figure S3 — Changes in interaction energies between individual residues of TRF1 and its surroundings (DNA and solvent combined) upon C1305 intercalation. Wide bars, colored according to amino acid type, show interaction energies in the absence of C-1305, while grey narrow bars correspond to respective energies after intercalation. In the simulation, the interacalating compound is inserted between the first and second GC pair in a GGG motif. Only the N-terminal linker and C-terminal helix regions are shown (cf. Fig. 5 in the main text). Note that strong repulsion of the rightmost Leu residue is due to its negatively charged C-terminal carboxyl group. (TIFF) [file pone.0089460.s003.tiff]

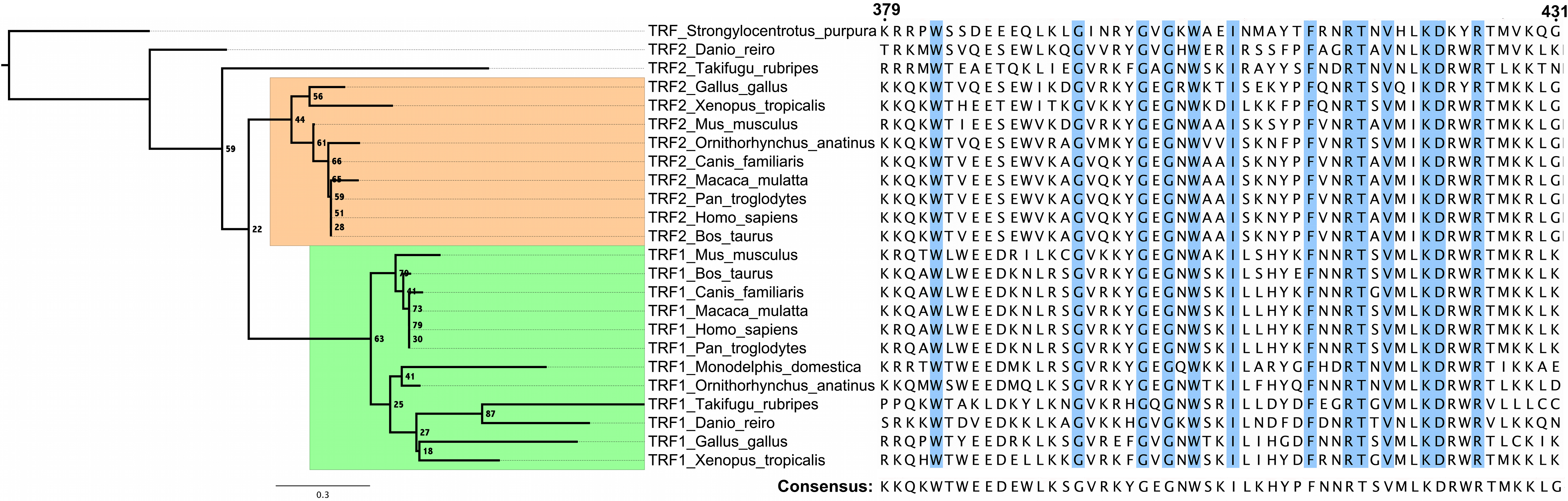

Supplement: Figure S4 — Evolution of Telomere-Repeat Binding Factors in vertebrates. Phylogenetic tree of vertabrate Telomeric Repeat Binding Factors calculated with RAxML and the alignment of DNA repet binding region with conserved amino acid positions marked in blue. (TIFF) [file pone.0089460.s004.tiff]

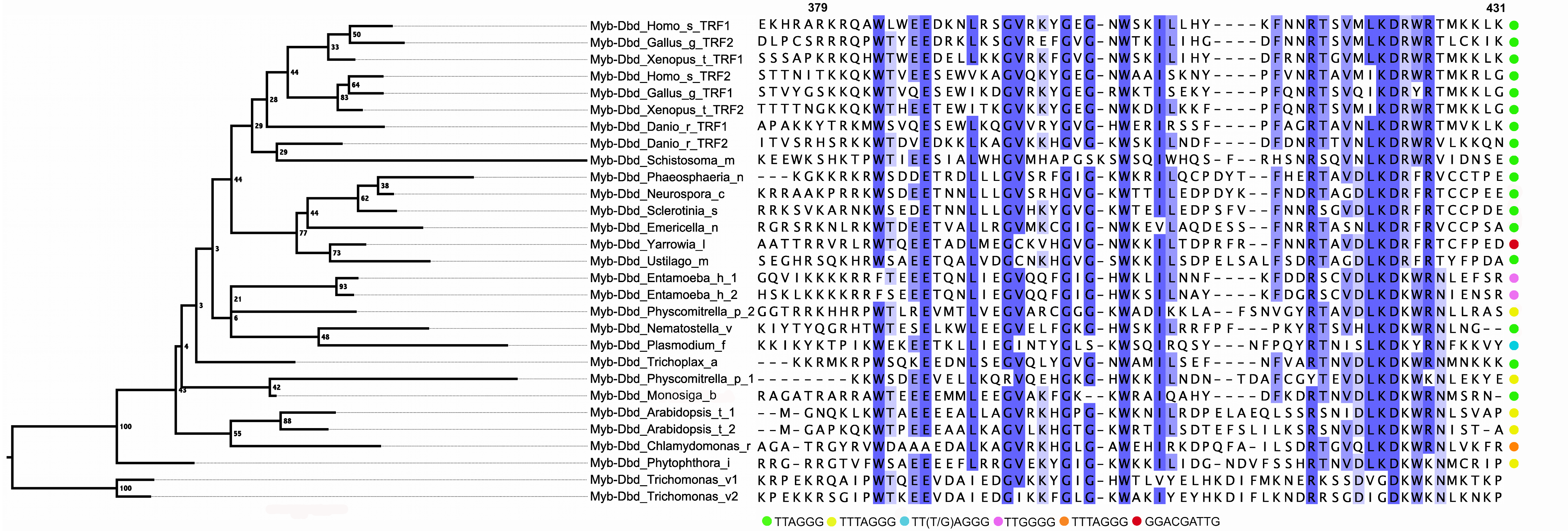

Supplement: Figure S5 — Evolution of Telomere-Repeat Binding Proteins in vertebrates. Phylogenetic tree of eukaryotic Telomeric Repeat Binding Proteins calculated with RAxML and the alignment of DNA repeat binding region with conserved amino acid positions marked in blue. On the right, colored dots correspond to different target sequences of the DNA-binding domains. (TIFF) [file pone.0089460.s005.tiff]

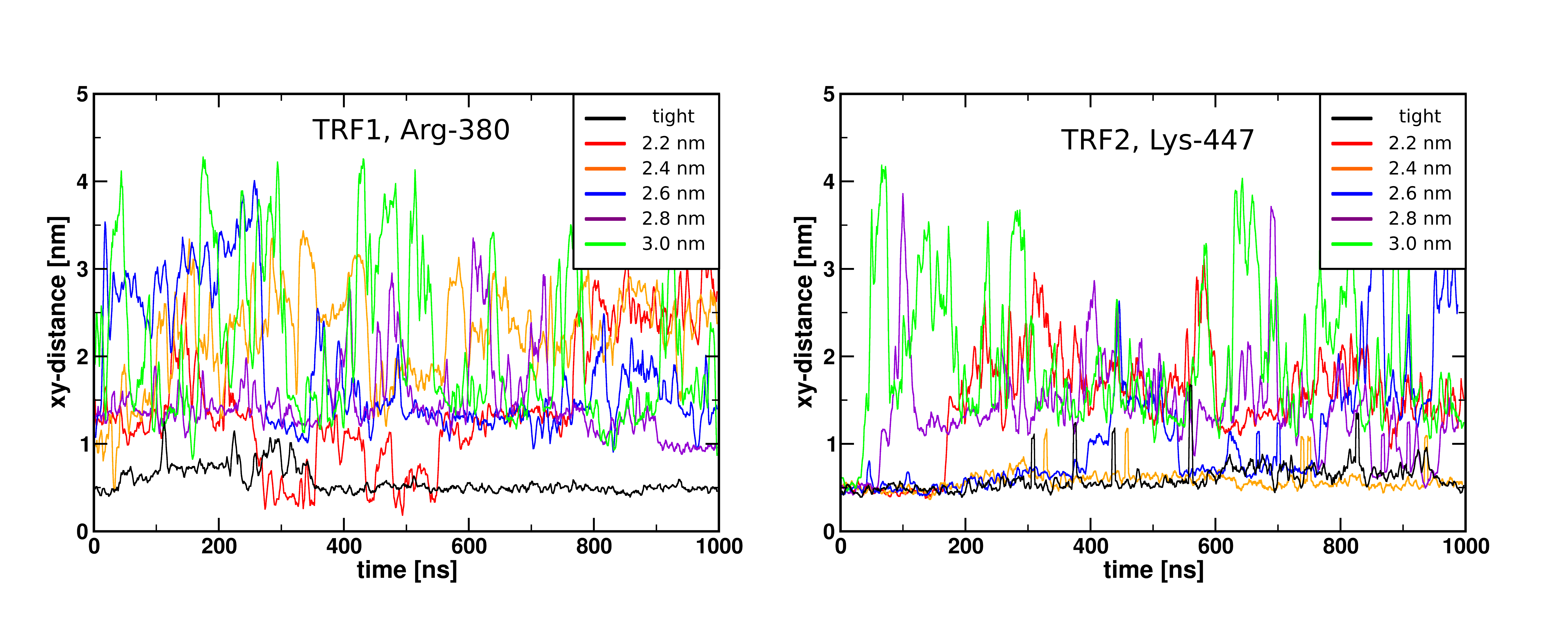

Supplement: Figure S6 — Dynamics of the flexible linker. Time series plots of xy-distance between the DNA and the linker for intermediate sampling windows (corresponding to reaction coordinate values from 2.2 to 3.0 nm) are shown to highlight sufficient sampling of the linker conformations in the study. The black line, corresponding to the tight complex, is shown for reference. In the plots, multiple binding and unbinding events can be seen, with noticeable baselines at ca. 0.5 nm (interactions with the minor groove) and ca. 1 nm (interactions with the DNA backbone). For distance calculation, a terminal side chain heteroatom of the respective residue was used as a reference group. (TIFF) [file pone.0089460.s006.tiff]

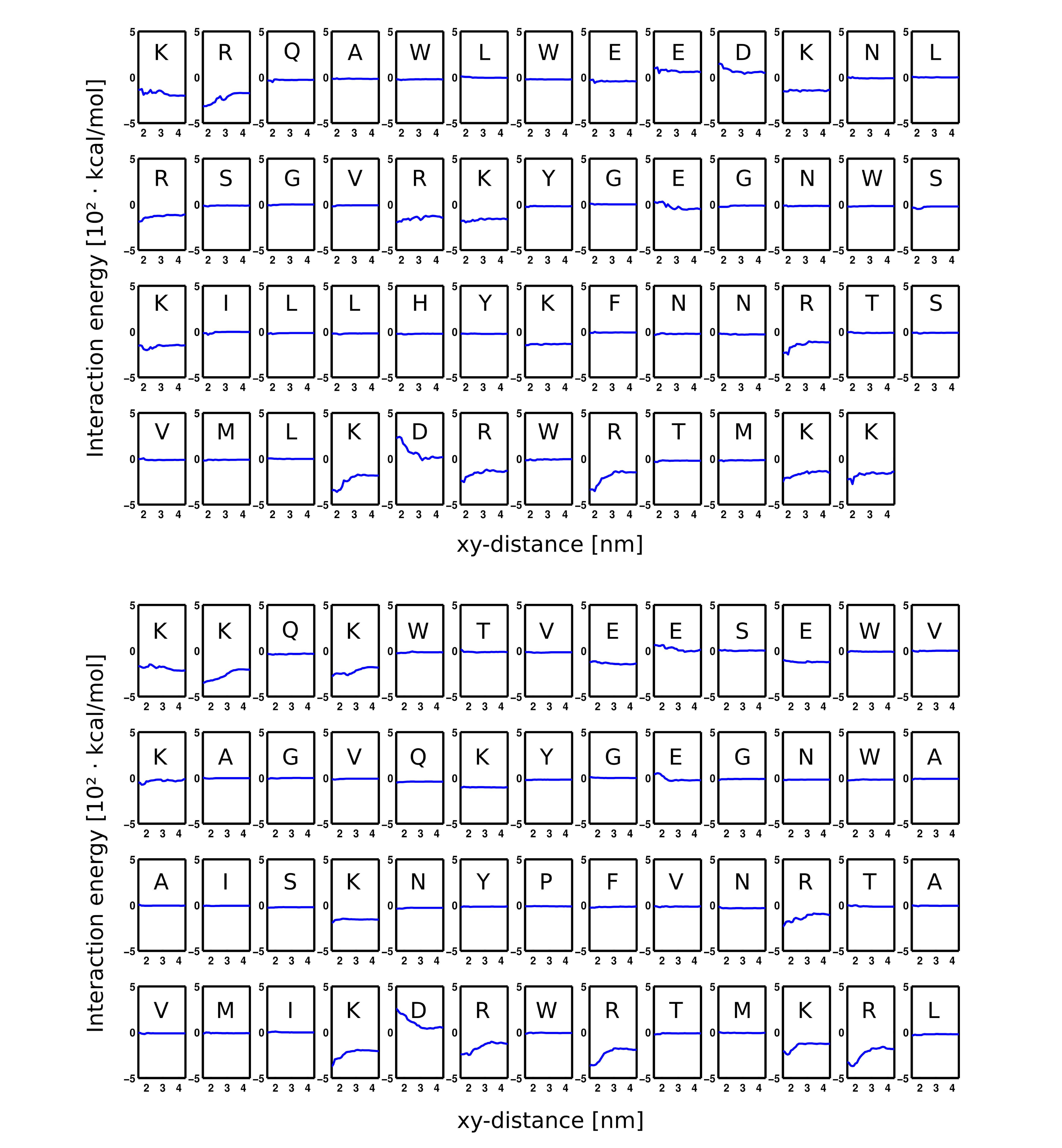

Supplement: Figure S7 — Interaction energy profiles for individual protein residues. Interaction energy (computed as a sum of electrostatic and van der Waals contributions) between all amino acid residues of TRF1 (top) and TRF2 (bottom) and their surroundings (DNA and solvent combined) as a function of the distance between the protein and the DNA axis. (TIFF) [file pone.0089460.s007.tiff]

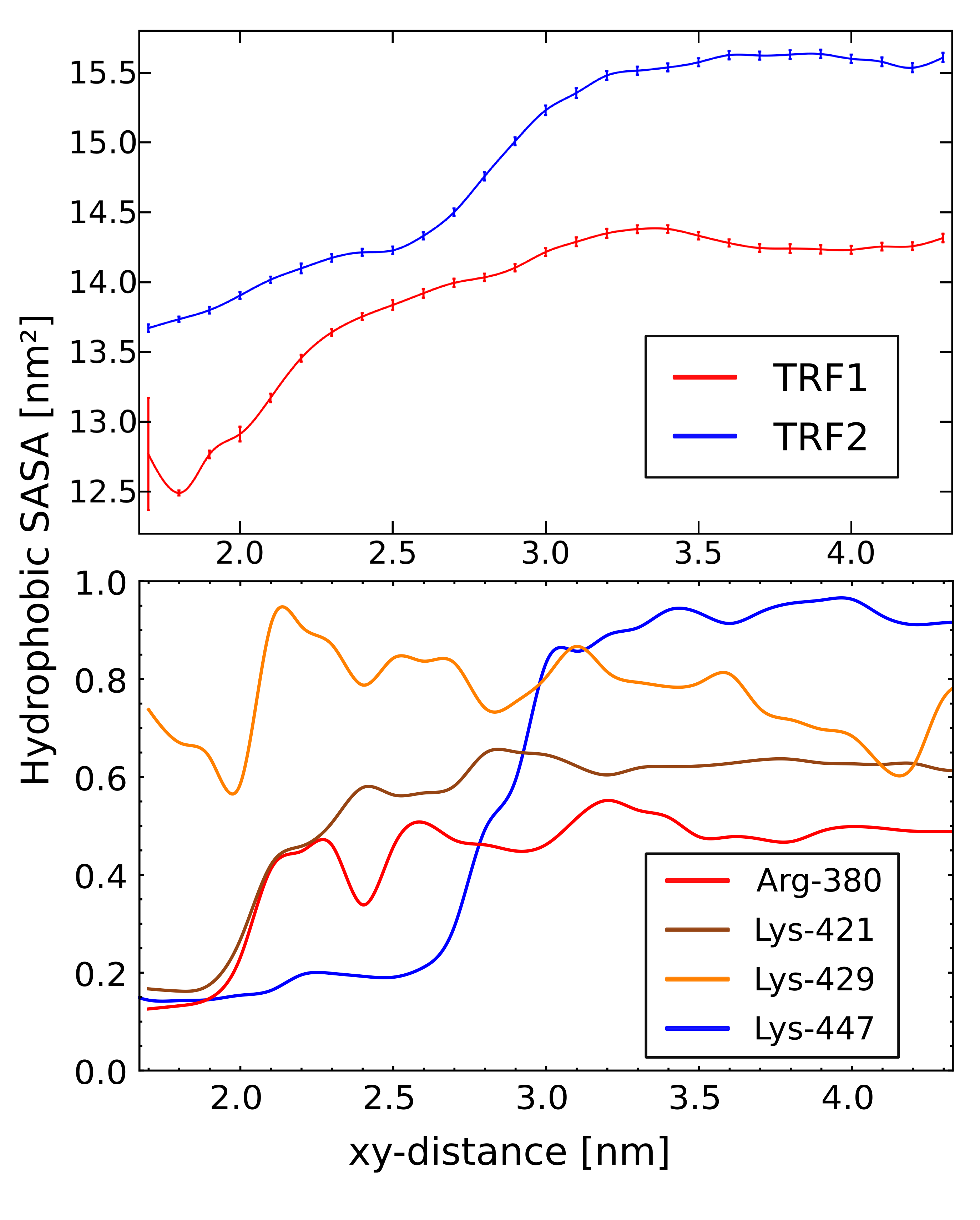

Supplement: Figure S8 — Hydrophobic solvent accessible surface area of TRF1 and TRF2 as a function of their distance from the DNA axis. The observed increase in the hydrophobic SASA upon dissociation (top) results mainly from the exposure of the three basic residues in TRF1 and one basic residue in TRF2 (bottom). (TIFF) [file pone.0089460.s008.tiff]
