# Supplementary material for: Molecular Recognition in Complexes of TRF Proteins with Telomeric DNA
Source: PLoS One. 2014 Feb 26;9(2):e89460. doi: 10.1371/journal.pone.0089460 (PMC3935891; doi:10.1371/journal.pone.0089460)
Supplement: File S1 — Tables S1–S8 present statistical data on direct and water-mediated hydrogen bonds between the TRF proteins and DNA. The results are sorted by protein (TRF1/TRF2), type of interaction (direct or water mediated hydrogen bond) and sequence-specificity (interaction with DNA bases or backbone). DNA base pairs are numbered consecutively as in the sequence 5′-GGTTAGGGTTAG-3′, with a prime symbol (’) indicating numeration of the complementary strand. (PDF) [file pone.0089460.s009.pdf]

Molecular Recognition in Complexes of TRF Proteins with Telomeric DNA

Miłosz Wieczór,[†] Adrian Tobiszewski,[†] Paweł Wityk,[†] Bartłomiej Tomiczek,[‡] and Jacek Czub[*,†]

Department of Physical Chemistry, Gdansk University of Technology,
ul. Narutowicza 11/12, 80-233 Gdansk, Poland, and
Department of Genetics, Evolution and Environment, University College London,
London, United Kingdom

E-mail: jacczub@pg.gda.pl

SUPPORTING INFORMATION

[*]To whom correspondence should be addressed
[†]Department of Physical Chemistry, Faculty of Chemistry, Gdansk University of Technology, Gdansk, Poland
[‡]Department of Genetics, Evolution and Environment, University College London, London, United Kingdom

Tables S1-S8 present statistical data on direct and water-mediated hydrogen bonds between the TRF proteins and DNA. The results are sorted by protein (TRF1/TRF2), type of interaction (direct or water mediated hydrogen bond) and sequence-specificity (interaction with DNA bases or backbone). DNA base pairs are numbered consecutively as in the sequence 5'-GGTTAGGGTTAG-3', with a prime symbol (') indicating numeration of the complementary strand.

Table S1: **Direct hydrogen bonds between TRF1 and the telomeric DNA nucleobases.**

Residues		Probability	Mean lifetime [ps]	Maximum lifetime [ps]
DNA	Protein			
G6	Lys-421	0.565	118.7 ± 2.9	4080
G7	Arg-425	0.889	235.3 ± 7.3	7000
C7'	Asp-422	0.519	162.7 ± 6.4	8420
C8'	Asp-422	0.577	235 ± 15	16260
A10'	Arg-380	0.437	169.7 ± 7.7	5720
T10	Arg-380	0.425	381 ± 60	24060
11A	Arg-380	0.077	36.6 ± 2.1	1610

Table S2: **Direct hydrogen bonds between TRF1 and the DNA sugar-phosphate backbone.**

Residues		Probability	Mean lifetime [ps]	Maximum lifetime [ps]
DNA	Protein			
T4	Ser-404	0.998	6090 ± 910	81310
T4	Ser-417	0.338	71.3 ± 3.8	8860
A5	Lys-421	0.250	608 ± 38	4960
A5	Trp-403	0.998	4280 ± 270	19590
G6	Lys-421	0.183	84.5 ± 2.3	1210
C7'	Lys-379	0.172	45.92 ± 0.97	780
C8'	Arg-415	0.103	52.8 ± 5.8	6660
C8'	Gln-381	0.594	43.6 ± 1.2	5600
A9'	Arg-423	0.969	366 ± 12	8180
A9'	Gln-381	0.440	29.41 ± 0.31	1130
A9'	Thr-426	0.516	279 ± 12	5440
A9'	Trp-383	0.293	81 ± 3	2390
A10'	Arg-380	0.178	21.62 ± 0.22	470
A10'	Lys-429	0.094	72.2 ± 2.5	910
T10	Arg-380	0.055	11.96 ± 0.07	70
12G	Arg-380	0.557	93.2 ± 2.2	2570
12G	Lys-379	0.367	60.48 ± 0.92	1530

Table S3: **Water-mediated hydrogen bonds between TRF1 and the telomeric DNA nucleobases.**

Residues		Probability	Mean lifetime [ps]	Maximum lifetime [ps]
DNA	Protein			
G6	Lys-421	0.101	38.5 ± 1.5	1180
C7’	Asp-422	0.092	16.57 ± 0.21	290
C7’	Val-418	0.082	17.45 ± 0.21	170
G7	Asp-422	0.078	38.7 ± 1.2	860
C8’	Asp-422	0.129	20.79 ± 0.27	270
G8	Asp-422	0.094	24.09 ± 0.45	340
A9’	Arg-380	0.211	20.93 ± 0.24	460
A9’	Asp-422	0.401	41.04 ± 0.69	1340
A9’	Thr-426	0.060	19.51 ± 0.31	210
A10’	Arg-380	0.053	12.86 ± 0.13	160
11A	Arg-380	0.066	13.39 ± 0.13	140
11T’	Arg-380	0.066	17.29 ± 0.32	410

Table S4: **Water-mediated hydrogen bonds between TRF1 and the DNA sugar-phosphate backbone.**

Residues		Probability	Mean lifetime [ps]	Maximum lifetime [ps]
DNA	Protein			
T3	Ser-404	0.532	26.07 ± 0.16	270
T4	Asn-402	0.706	70.25 ± 0.92	1480
T4	Gly-401	0.139	13.17 ± 0.07	120
T4	Lys-405	0.072	18.36 ± 0.26	380
T4	Ser-417	0.301	31.11 ± 0.35	520
A5	Glu400	0.120	19.06 ± 0.30	410
A5	Gly-401	0.081	12.53 ± 0.08	130
G6	Arg-425	0.159	33.55 ± 0.78	1370
G6	Lys-421	0.081	28.53 ± 0.56	310
C7’	Lys-379	0.112	14.83 ± 0.12	150
G7	Arg-425	0.127	18.19 ± 0.23	700
C8’	Arg-380	0.066	13.48 ± 0.12	160
C8’	Arg-415	0.312	24.98 ± 0.31	750
C8’	Gln-381	0.589	53.98 ± 0.78	2140
C8’	Lys-379	0.342	21.70 ± 0.15	280
C8’	Trp-383	0.150	17.56 ± 0.18	280
A9’	Arg-380	0.190	20.98 ± 0.18	200
A9’	Arg-423	0.286	21.70 ± 0.17	240
A9’	Asp-422	0.329	31.66 ± 0.52	1400
A9’	Thr-426	0.226	39.0 ± 1.1	1360
A9’	Trp-383	0.484	28.41 ± 0.30	860
A10’	Arg-423	0.255	25.60 ± 0.31	440
A10’	Lys-428	0.066	18.36 ± 0.31	400
A10’	Lys-429	0.088	19.42 ± 0.25	180
A10’	Thr-426	0.279	29.29 ± 0.35	580
11A	Ace-378	0.084	19.72 ± 0.25	160
11A	Arg-380	0.165	21.65 ± 0.21	220
11A	Lys-379	0.156	22.81 ± 0.24	220
11T’	Lys-429	0.083	20.65 ± 0.29	200
12G	Ace-378	0.164	18.44 ± 0.15	180
12G	Arg-380	0.452	25.21 ± 0.21	900
12G	Lys-379	0.170	21.16 ± 0.25	310

Table S5: **Direct hydrogen bonds between TRF2 and the telomeric DNA nucleobases.**

Residues		Probability	Mean lifetime [ps]	Maximum lifetime [ps]
DNA	Protein			
A5	Lys-488	0.485	44.90 ± 0.55	830
G6	Lys-488	0.316	45.2 ± 1.2	2950
G7	Arg-492	0.962	419 ± 14	8770
C7’	Asp-489	0.606	111.6 ± 3.3	5820
C8’	Asp-489	0.839	243.2 ± 7.3	10470
A9’	Lys-447	0.143	23.33 ± 0.42	690
T10	Lys-447	0.634	38.95 ± 0.34	860
11A	Lys-447	0.212	17.07 ± 0.15	270

Table S6: **Direct hydrogen bonds between TRF2 and the DNA sugar-phosphate backbone.**

Residues		Probability	Mean lifetime [ps]	Maximum lifetime [ps]
DNA	Protein			
T4	Ala-471	0.839	86.4 ± 1.1	1820
T4	Trp-470	0.105	15.11 ± 0.18	360
A5	Trp-470	0.996	2740 ± 160	18090
C7’	Lys-446	0.070	55.0 ± 1.7	530
C8’	Gln-448	0.577	39.80 ± 0.91	3530
C8’	Trp-450	0.215	41.0 ± 1.1	1580
A9’	Arg-490	0.879	272.1 ± 7.2	4090
A9’	Gln-448	0.589	37.39 ± 0.38	810
A9’	Lys-449	0.144	40.5 ± 1.2	2900
A9’	Thr-493	0.188	101.3 ± 5.9	6090
A9’	Trp-450	0.687	358 ± 22	12460
A10’	Arg-496	0.732	1350 ± 120	22440
A10’	Lys-449	0.057	20.01 ± 0.31	160
11A	Lys-447	0.405	23.44 ± 0.16	310
12G	Lys-446	0.377	64.50 ± 0.99	710
12G	Lys-447	0.714	64.39 ± 0.92	2470

Table S7: **Water-mediated hydrogen bonds between TRF2 and the telomeric DNA nucleobases.**

Residues		Probability	Mean lifetime [ps]	Maximum lifetime [ps]
DNA	Protein			
A5	Lys-488	0.242	18.27 ± 0.13	230
G6	Asp-489	0.242	22.91 ± 0.21	240
G6	Lys-488	0.178	16.78 ± 0.15	550
C7’	Asp-489	0.077	16.30 ± 0.27	360
C7’	Val-485	0.140	19.43 ± 0.18	170
G7	Asp-489	0.072	33.0 ± 1.2	800
C8’	Asp-489	0.061	20.07 ± 0.35	200
C8’	Lys-447	0.074	24.68 ± 0.47	360
G8	Arg-496	0.134	39.26 ± 0.71	350
A9’	Arg-496	0.183	18.15 ± 0.15	280
A9’	Asp-489	0.257	37.51 ± 0.78	1600
A9’	Thr-493	0.112	19.19 ± 0.23	260
A10’	Lys-447	0.051	21.48 ± 0.44	290

Table S8: **Water-mediated hydrogen bonds between TRF2 and the DNA sugar-phosphate backbone.**

Residues		Probability	Mean lifetime [ps]	Maximum lifetime [ps]
DNA	Protein			
T3	Lys-475	0.273	22.20 ± 0.16	230
T4	Ala-484	0.172	15.04 ± 0.09	140
T4	Asn-469	0.511	33.20 ± 0.31	710
T4	Gly-468	0.172	14.13 ± 0.08	120
A5	Glu467	0.327	25.83 ± 0.30	610
G6	Lys-495	0.059	14.81 ± 0.17	130
C7’	Lys-446	0.121	14.92 ± 0.12	150
G7	Arg-492	0.102	17.05 ± 0.16	140
C8’	Arg-482	0.654	58.03 ± 0.72	1240
C8’	Gln-448	0.287	29.54 ± 0.48	940
C8’	Lys-446	0.445	23.66 ± 0.16	330
C8’	Lys-447	0.145	16.98 ± 0.14	160
C8’	Trp-450	0.104	18.24 ± 0.33	650
A9’	Arg-490	0.246	35.04 ± 0.62	1160
A9’	Asp-489	0.113	27.55 ± 0.92	970
A9’	Lys-449	0.061	16.11 ± 0.22	270
A9’	Thr-493	0.595	132.6 ± 4.6	4460
A9’	Trp-450	0.495	33.74 ± 0.30	370
A10’	Arg-496	0.217	18.15 ± 0.17	430
A10’	Lys-449	0.067	16.27 ± 0.22	190
A10’	Thr-493	0.122	25.99 ± 0.47	750
11A	Ace-445	0.200	20.76 ± 0.18	210
11A	Lys-446	0.083	21.31 ± 0.27	130
11T’	Arg-496	0.072	30.83 ± 0.73	280
12G	Ace-445	0.285	27.70 ± 0.24	250
12G	Lys-446	0.127	16.71 ± 0.17	320
12G	Lys-447	0.543	26.96 ± 0.17	430
12G	Lys-449	0.066	12.90 ± 0.09	80
